# “Like Recycles Like”: Selective Ring‐Closing Depolymerization of Poly(L‐Lactic Acid) to L‐Lactide

**DOI:** 10.1002/anie.202204531

**Published:** 2022-06-09

**Authors:** Linnea Cederholm, Jakob Wohlert, Peter Olsén, Minna Hakkarainen, Karin Odelius

**Affiliations:** ^1^ Wallenberg Wood Science Center, WWSC Department of Fibre and Polymer Technology KTH Royal Institute of Technology Teknikringen 56–58 100 44 Stockholm Sweden

**Keywords:** Chemical Recycling, L-Lactide, Ring-Opening Polymerization, Solvent Effects, Thermodynamics

## Abstract

Chemical recycling of poly(L‐lactic acid) to the cyclic monomer L‐lactide is hampered by low selectivity and by epimerization and elimination reactions, impeding its use on a large scale. The high number of side reactions originates from the high ceiling temperature (*T*_c_) of L‐lactide, which necessitates high temperatures or multistep reactions to achieve recycling to L‐lactide. To circumvent this issue, we utilized the impact of solvent interactions on the monomer–polymer equilibrium to decrease the *T*_c_ of L‐lactide. Analyzing the observed *T*_c_ in different solvents in relation to their Hildebrand solubility parameter revealed a “like recycles like” relationship. The decreased *T*_c_, obtained by selecting solvents that interact strongly with the monomer (dimethyl formamide or the green solvent γ‐valerolactone), allowed chemical recycling of high‐molecular‐weight poly(L‐lactic acid) directly to L‐lactide, within 1–4 h at 140 °C, with >95 % conversion and 98–99 % selectivity. Recycled L‐lactide was isolated and repolymerized with high control over molecular weight and dispersity, closing the polymer loop.

## Graphical abstract

A novel approach is taken to decrease the ceiling temperature of L‐lactide, enabling chemical recycling of high molecular weight poly(L‐lactic acid) directly back to the cyclic lactide monomer (CRM) with high selectivity. By utilizing specific solvent interactions, the CRM can be performed at a moderate temperature; thereby, obstructing side reactions are diminished.

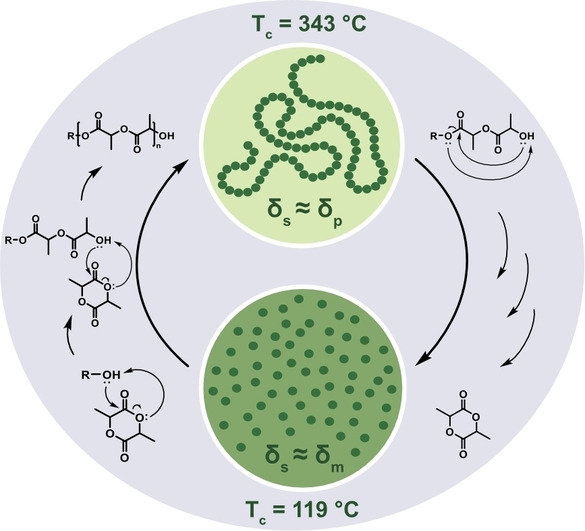

## Introduction

The pursuit of sustainability within the polymer industry must include efficient waste management and recycling pathways.^[1]^ Though mechanical recycling should be the first choice when suitable, deterioration in physical properties upon repeated reprocessing limits the scope.[^1a^, ^1b^, ^1d^] Chemical recycling to monomer (CRM) has, therefore, been emphasized as the best option to achieve a truly circular polymer economy, despite limitations dictated by the polymers’ chemical structure and technological challenges that still need to be overcome.[^1c^, ^1d^] One interesting group of polymers for CRM includes those synthesized through ring‐opening polymerization (ROP) of heterocyclic monomers. The inherent reversibility of ROP equilibrium reactions can be utilized for CRM when proper circumstances are created. The monomer‐polymer equilibrium is determined by the change in enthalpy (Δ*H*_p_) and entropy (Δ*S*_p_) upon polymerization, in combination with the system temperature and concentration.^[2]^ The relationship between Δ*H*_p_ and Δ*S*_p_ determines the ceiling temperature (*T*_c_), i.e., the temperature above which the monomer‐polymer equilibrium is pushed completely toward monomer formation, yet *T*_c_ is also concentration dependent. Δ*H*_p_ and Δ*S*_p_ are governed by the ring size, heteroatoms in the ring and ring substituents,^[3]^ and the monomers most attractive for CRM are those that can be polymerized at high conversion but also have a low to moderate *T*_c_. Examples of such monomers, where both polymerization and depolymerization have been demonstrated, include β‐methyl‐δ‐valerolactone,^[4]^ δ‐caprolactone,^[5]^ γ‐butyrolactone,^[6]^ 4,5‐*trans*‐hexahydro‐2(3*H*)‐benzofuranone,^[7]^ 3,4‐*trans*‐hexahydro‐2(3*H*)‐benzofuranone,^[8]^ 2,3‐dihydro‐5*H*‐1,4‐benzodioxepin‐5‐one,^[9]^ and 1‐benzyloxycarbonyl‐3,4‐epoxy pyrrolidine copolymerized with CO_2_.^[10]^ However, the corresponding polymers have, thus far, no commercial impact.

**Figure 1:**
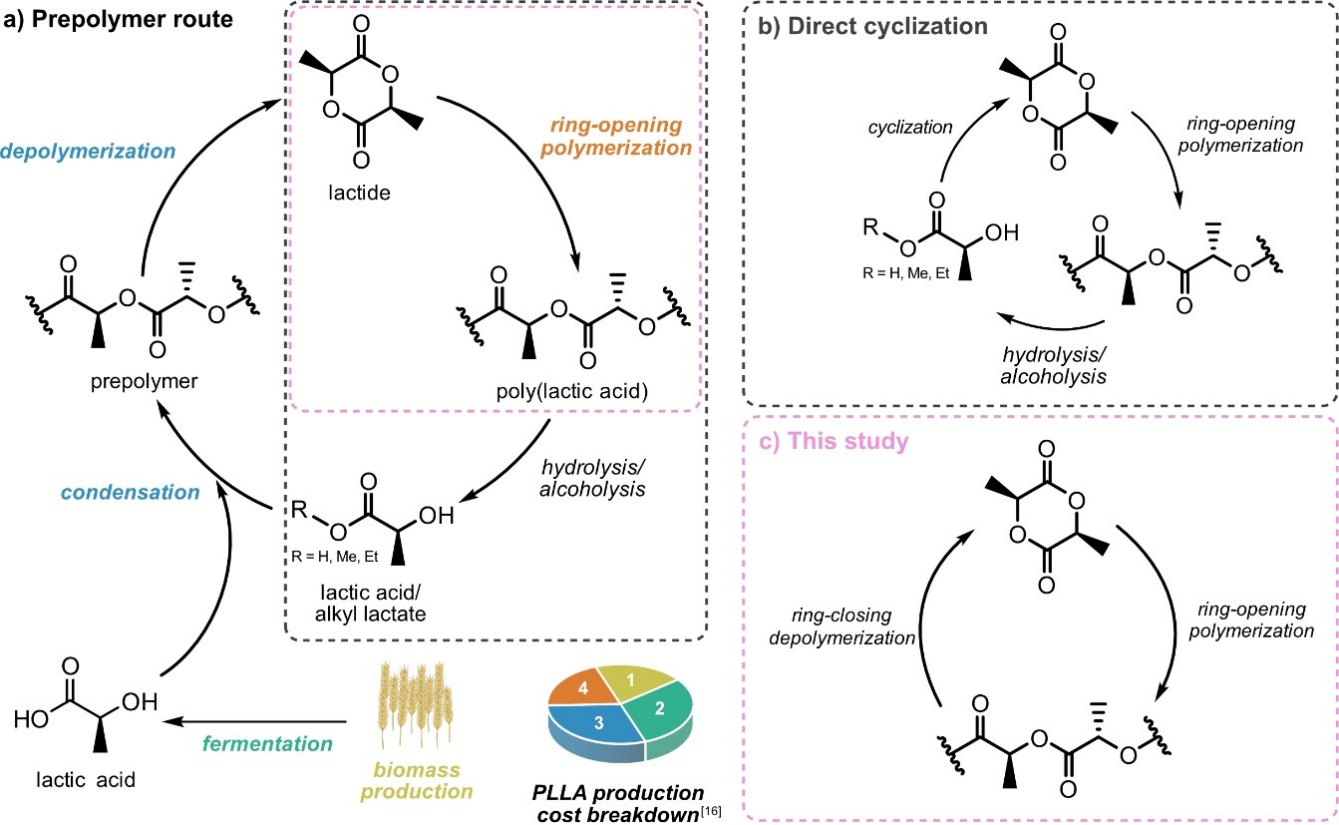
Life cycle of PLLA. a) Production of PLLA from biomass and chemical recycling via the prepolymer route. The estimated cost required for each production step is illustrated in the PLLA production breakdown: 1) biomass production; 2) production of lactic acid via fermentation; 3) LLA production, including condensation oligomerization and subsequent depolymerization; and 4) ring‐opening polymerization.^[16]^ b) Chemical recycling via direct cyclization of lactic acid or alkyl lactates to LLA. c) CRM via one‐step ring‐closing depolymerization of PLLA to LLA.

Poly(lactic acid) (PLA) is currently the most utilized commercial biobased synthetic polymer.^[11]^ It is synthesized on a large scale utilizing a multistep methodology, where lactic acid is first oligomerized through step‐growth condensation (average molecular weight of prepolymer: 400–2500 g mol^−1^),^[12]^ followed by depolymerization to lactide (LA) and subsequent ROP of LA into high molecular weight PLA (Figure 1a). PLA is biodegradable under certain conditions, and is often marketed as such.^[13]^ However, the time scale for complete biodegradation, where biomass, CO_2_, CH_4_ and H_2_O are the sole products, depends on the environment, e.g. temperature, humidity and microorganisms.^[13a]^ Hence, PLA only passes international biodegradation standards in controlled industrial environments where the temperature is above 50 °C.^[14]^ Even so, it is important that also biodegradable polymers are recyclable, and the European Union strategy towards 100 % recyclable or reusable plastic by 2030^[15]^ is one driving force. In addition, approximately half of the costs during PLA production arise from lactic acid manufacturing, which includes the production of biomass (yellow and green process steps in Figure 1a),^[16]^ an economic factor that further motivates closing the loop for PLA. Mechanical recycling of PLA is viable with proper sorting technology, though often requiring blending with at least 50 % virgin material.[^17^, ^18^] When mechanical recycling is no longer possible, chemical recycling to produce new PLA, is preferred to virgin PLA derived from biomass, why efficient CRM strategies are needed.^[19]^

High molecular weight PLA can be chemically recycled via hydrolysis or alcoholysis to lactic acid or alkyl lactates, which subsequently can be resubmitted into the prepolymer route.^[20]^ This methodology is applied by NatureWorks to scrap PLA^[20a]^ but has also been demonstrated for postconsumer PLA waste containing impurities of poly(ethylene terephthalate) and polypropylene.^[20b]^ Direct cyclization of lactic acid^[21]^ or alkyl lactates^[22]^ are other interesting alternatives, reducing the number of reaction steps from four to three (Figure 1b). An even more appealing procedure would be to, in one step, depolymerize PLA directly to LA through ring‐closing depolymerization, thereby decreasing the number of reaction steps to a minimum (Figure 1c). This path is less viable for LA than for, e.g., γ‐butyrolactone since the temperature needed to push the monomer‐polymer equilibrium toward complete monomer formation in bulk is much higher for LA (*T*_c_>600 °C)^[23]^ than for γ‐butyrolactone (*T*_c_=22 °C). Side reactions, such as epimerization to a mixture of L‐lactide (LLA), D‐lactide (DLA) and *meso*‐LA, elimination reactions generating acrylic acid and acrylic oligomers, and intramolecular transesterification resulting in cyclic polymer formation, are consequences of the high temperature that is required.^[24]^ Although the presence of tin(II) ethylhexanoate (Sn(Oct)_2_) during thermal decomposition can render depolymerization selective toward cyclic monomers,^[24]^ these side reactions are problematic and delimit the efficiency of CRM to pure LA.^[25]^ In LA production, this issue is resolved by a constant removal of formed LA from the system by vacuum distillation, enabling depolymerization of the prepolymer to proceed, even though polymer formation is thermodynamically favored. Hence, temperatures far below *T*_c_, approximately 200 °C, can be utilized.^[26]^ However, even at these decreased temperatures, epimerization reactions occur to a significant degree, resulting in crude LLA with a *meso*‐LA content of 4–11 % at only 73 % conversion of the prepolymer.^[12]^ Together with elimination reactions generating acrylic acid, these side reactions affect the purity of the crude LA formed and subsequently the final yield.[^26a^, ^27^] As stated, the high recycling temperature for PLA is inherent to the high *T*_c_ of LA. Hence, if the *T*_c_ value could be lowered, this could enable CRM at lower temperatures.

It is well established that *T*_c_ is concentration dependent, where a decreased concentration results in a decreased *T*_c_. Less direct is the effect that different solvent properties have on Δ*H*_p_ and Δ*S*_p_ and thus also on *T*_c_. In the 1970s, Ivin and Léonard used Flory–Huggins solubility theory^[28]^ to describe how monomer‐polymer equilibrium is influenced by interactions between monomers, polymers and solvents.^[29]^ Although there are examples in the literature that report on this solvent effect on the equilibrium polymerization of α‐methylstyrene,^[30]^ tetrahydrofuran,^[31]^ and cyclic carbonate 2‐allyloxymethyl‐2‐ethyl‐trimethylene carbonate,^[32]^ it is not commonly taken into account when presenting thermodynamic polymerization data or when choosing the appropriate solvent for a reaction.^[33]^ While dilution can be utilized for controlled ring‐closing depolymerization to monomers with low to moderate *T*_c_ (bulk), the initial *T*_c_ of LA in bulk is too high to be efficiently decreased by merely dilution in nonpolar solvents such as toluene.^[9]^ However, a solvent that interacts more strongly with the monomer might facilitate more effective *T*_c_ depression.

We therefore considered whether the *T*_c_ of LA could be decreased enough to eliminate the need for LA distillation by introducing a polar solvent into the system. To achieve this milder and less demanding setup, the *T*_c_ must be decreased to a temperature at which complete depolymerization can be practically performed while side reactions are limited. Therefore, the two main questions that we asked ourselves were i) Can a conventional solvent decrease the *T*_c_ of LA enough to make CRM feasible? and ii) What are the controlling factors behind the solvent's effect on *T*_c_?

## Results and Discussion

**Figure 2:**
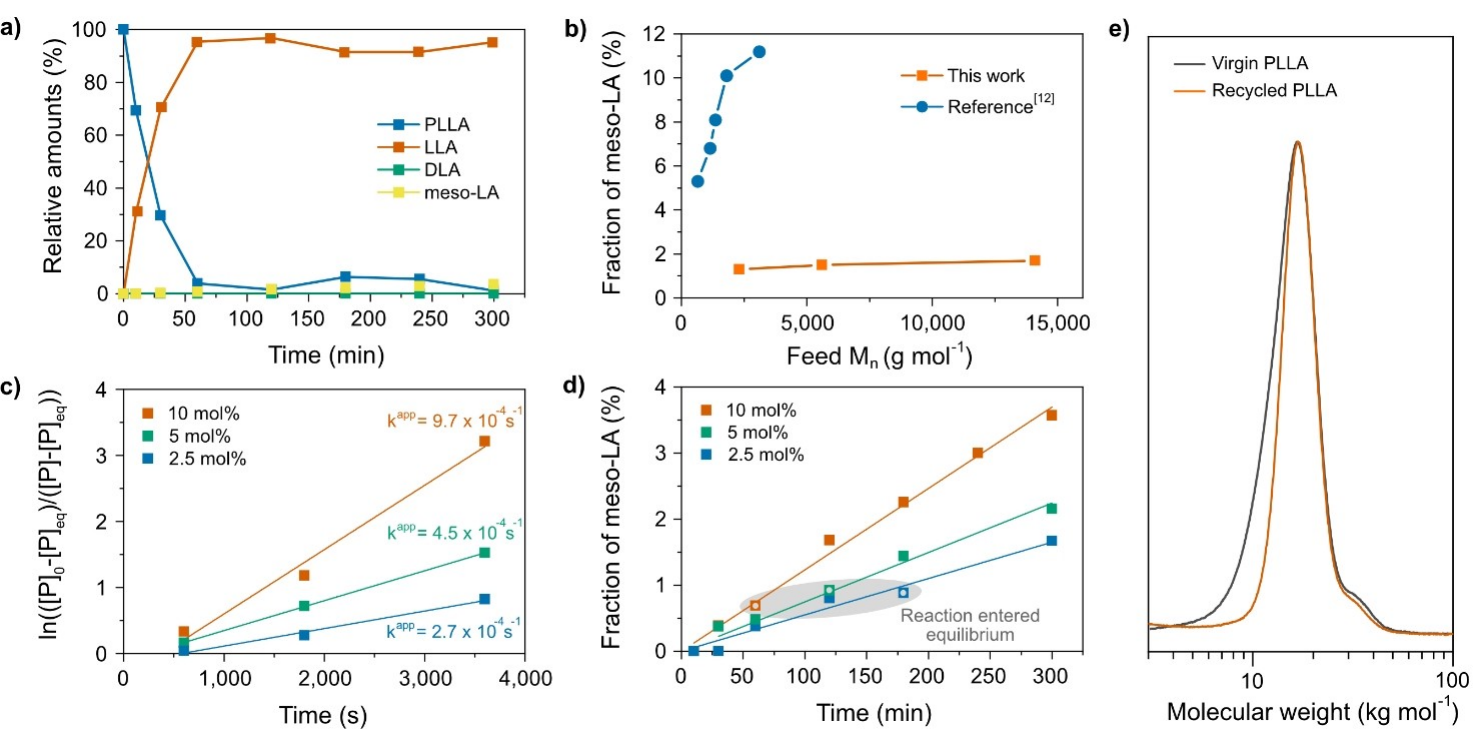
Depolymerization of PLLA and repolymerization of recycled LLA. a) Kinetics of solution depolymerization of PLLA (0.5 M based on the repeating unit of LLA; *M*_n_=14 000 g mol^−1^) in DMF at 140 °C with 10 mol % Sn(Oct)_2_ as the catalyst. The relative amounts of PLLA polymer/oligomer, LLA, DLA and *meso*‐LA were calculated from ^1^H‐NMR data. b) Fraction of *meso*‐LA (Equation (SE1) in Supporting Information Note 1) in crude lactide in relation to feed molecular weight. Solution depolymerization of PLLA (0.5 M based on the repeating unit of LLA; *M*_n_=14 000 g mol^−1^) in DMF at 140 °C with 10 mol % Sn(Oct)_2_ as the catalyst after a 2 h reaction ( 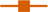). Values from reference for continuous distillation ( 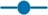).^[12]^ c) Reaction rate (*k* ^app^) in relation to the Sn(Oct)_2_ concentration for depolymerization of PLLA (0.5 M based on the repeating unit of LLA; *M*_n_=14 000 g mol^−1^) in DMF at 140 °C. d) Fraction of *meso*‐LA in crude lactide over time in relation to the Sn(Oct)_2_ concentration for depolymerization of PLLA (0.5 M based on the repeating unit of LLA; *M*_n_=14 000 g mol^−1^) in DMF at 140 °C. The data points marked with correspond to the fraction of *meso*‐LA at the time the reaction entered equilibrium. e) SEC chromatogram of virgin PLLA ( 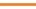) and PLLA synthesized from purified LLA recycled via solution depolymerization ( 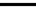). The feed ratio was [LLA] : [BnOH] : [Sn(Oct)_2_]=100 : 1 : 1 for both polymerizations.

The working hypothesis was that a solvent with high polarity may depress the *T*_c_ of LA, enabling CRM of PLA in solution. The first experiment was therefore carried out in dimethylformamide (DMF), a highly polar aprotic solvent, with 0.5 M (based on the repeating unit of LA) poly(L‐lactic acid) (PLLA, i.e. PLA synthezised from 100 % L‐lactide; *M*_n_=14 000 g mol^−1^, *Ð*=1.13; Supporting Information Figure 1) and 10 mol % Sn(Oct)_2_ as the catalyst. The reaction temperature was set to 10 degrees below the boiling point of DMF, that is, 140 °C, and the stability of DMF under these conditions was confirmed (Supporting Information Figure 3). Gratifyingly, the depolymerization was rapid and reached 96 % conversion to cyclic monomer within 1 h (Figure 2a, Supporting Information Figure 4, Supporting Information Table 2.1 and Supporting Information Note 1). End‐group analysis by ^1^H‐NMR spectroscopy depicted a linear relationship between molecular weight and conversion (Supporting Information Figure 5), which indicates that the ring‐closing depolymerization took place through an “unzipping” mechanism from the −OH functional propagating chain end. The proposed depolymerization mechanism was also supported by depolymerization experiments of end capped PLLA (Supporting Information Figure 6). The diastereomeric purity of the generated monomer was high (1 h: 99.3 % LLA, 0.7 % *meso*‐LA, ≪0.1 DLA; Equations (SE1–SE3) in Supporting Information Note 1) but decreased slightly over time (5 h: 96.4 % LLA, 3.6 % *meso*‐LA, <0.1 DLA) due to epimerization. These results were intriguing, as one constraining factor for CRM of PLA is the high degree of epimerization.^[1d]^ In a patent describing the production of LLA via a prepolymer route through a continuous distillation process, an increased *meso*‐LA content of the crude LLA product with an increased molecular weight of the PLLA feed was reported.^[12]^ Through continuous distillation (10 mm Hg and 200–220 °C) to approximately 73 % conversion to monomer, the *meso*‐LA content increased from 5.3 % to 11 % as the feed average molecular weight was increased from 640 g mol^−1^ to 3100 g mol^−1^. To compare these values to the solution depolymerization demonstrated in this work, two additional batches of PLLA with different molecular weights were synthesized and depolymerized under the same conditions as described above (*M*_n_=2300 g mol^−1^, *Ð*=1.21; *M*_n_=5700 g mol^−1^, *Ð*=1.14; Supporting Information Figure 1). Compared to the values reported for the continuous distillation process,^[12]^ the changes in diastereomeric purity as the feed *M*_n_ was increased from 2300 g mol^−1^ to 14 000 g mol^−1^ were negligible, and epimerization in the solution depolymerization process appeared independent of feed molecular weight (Figure 2b, Supporting Information Tables 2.1–2.3). Another factor that affects the degree of epimerization, and hence the diastereomeric purity of the generated LLA, is the catalyst concentration. The amount of Sn(Oct)_2_ was therefore decreased to 5 mol % and 2.5 mol %, and depolymerizations were carried out as described before. As expected, the rate of depolymerization decreased with decreasing catalyst concentration (Figure 2c, Supporting Information Tables 2.1 and 2.4–2.5), and it had a significant effect on epimerization with time; however, as the reaction approached equilibrium, the diastereomeric purity was similar (Figure 2d).

Although the feed molecular weight had a negligible effect on the diastereomeric purity of the generated LLA, a lower feed molecular weight led to a slightly negative effect on the equilibrium conversion (85 % for *M*_n_=2300 g mol^−1^). This result is associated with the influence that the degree of polymerization has on the monomer‐polymer equilibrium, which starts to be apparent at lower molecular weights (Supporting Information Note 3).^[34]^ Hence, one can expect solution depolymerization to also be effective for high molecular weight PLA, and we demonstrated this for commercial grade PLA (*M*_n_=110 000 g mol^−1^, *Ð*=2.02; Supporting Information Figure 1) in DMF at 140 °C with 10 mol % Sn(Oct)_2_ as the catalyst (Supporting Information Table 2.6). The reaction rate (*k* ^app^=9.6×10^−4^ s^−1^) was comparable to that of depolymerization with the same catalyst concentration and a feed molecular weight of 14 000 g mol^−1^ (*k* ^app^=9.7×10^−4^ s^−1^), and 96 % polymer‐to‐monomer conversion was reached within 1 h. Commercial PLA is generally synthesized from LLA with a few percent of DLA or *meso*‐LA, here, a DLA content of approximately 3–5 % was reported, and the diastereomeric purity of the LLA generated from depolymerization of commercial PLA was, therefore, expected to decrease compared to that of pure PLLA. Indeed, the fraction of *meso*‐LA in crude lactide (Equation (SE1) in Supporting Information Note 1) at 96 % conversion was 6.3 %, which is 9‐fold higher than that of pure PLLA. However, the fraction of *meso*‐LA at 23 % conversion was already 5.7 %, compared to <0.1 % at 31 % conversion for pure PLLA (*M*_n_=14 000 g mol^−1^) synthesized in the laboratory. This observation suggests that a majority of the generated *meso*‐LA does not arise from epimerization during depolymerization but rather from optical irregularities in the feed PLA.

Next, we looked into the repolymerizability of the generated monomer. First, we could conclude that the monomer was active towards ROP and formed, as expected, PLLA oligomers. This was demonstrated by adding 10 mol % of TBD to the crude LLA‐DMF solution (PLLA: *M*_n_=14 000 g mol^−1^, *Ð*=1.13) at room temperature (Supporting Information Figure 7). However, to obtain control over the molecular weight, the monomer must be isolated and purified. This was achieved by distillation of DMF (84 % yield, 99.9 % purity) from the crude LA (PLA: *M*_n_=110 000 g mol^−1^, *Ð*=2.02) followed by azeotropic distillation with n‐heptane and subsequent recrystallization in toluene (Supporting Information Figure 8). The purified monomer (38 % yield; 3 % *meso*‐LA content) was used subsequently in ROP to PLA, with the same high control over molecular weight and dispersity as for ROP of virgin LLA (Figure 2e). Isolation and purification of the recycled LA is indeed a challenging step which here was not optimized. Nevertheless, these results demonstrate CRM of PLA via direct ring‐closing depolymerization and subsequent repolymerization, closing the polymer loop.

**Figure 3:**
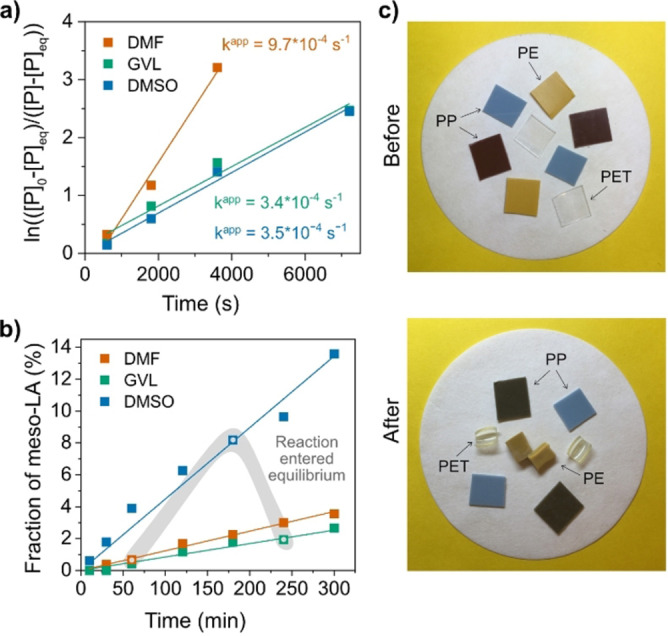
Depolymerization of PLLA in DMF, GVL and DMSO at 140 °C. Initial PLLA concentration: 72 mg mL^−1^ (corresponds to [M]=0.5 M at complete depolymerization to lactide). Feed PLLA: *M*_n_=14 000 g mol^−1^. Sn(Oct)_2_ concentration: 10 mol %. a) Reaction rate (*k* ^app^) in relation to reaction medium. b) Fraction of *meso*‐LA (Equation (SE1) in Supporting Information Note 1) in crude lactide over time in relation to reaction medium. The data points marked with 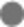 correspond to the fraction of *meso*‐LA at the time the reaction entered equilibrium. c) Chemical recycling of PLLA in the presence of mixed plastic waste. The image shows PLLA, PE (yellow), PP (brown and blue) and PET pieces from postconsumer plastic (Supporting Information Figure 10). Top: plastic pieces before depolymerization of PLLA. Bottom: insoluble fraction after depolymerization of PLLA and subsequent filtration.

At this point, the depolymerization has been studied with DMF as solvent. We anticipated that the depolymerization was promoted by the high polarity of DMF and, therefore, we decided to also evaluate dimethyl sulfoxide (DMSO) and γ‐valerolactone (GVL). DMSO is more polar than DMF, whereas GVL is less polar. DMSO and GVL lack the toxic and hazardous properties of DMF, and they are classified as nonharmful substances. Moreover, GVL is a green solvent produced from cellulose or hemicellulose and has the potential to replace DMF and *N*‐methyl‐pyrrolidone (NMP), both of which are reprotoxic.^[35]^ Initially, the reactions were performed at 140 °C with 0.5 M PLLA and 10 mol % Sn(Oct)_2_ (Supporting Information Tables 2.7 and 2.8). The reaction rates in GVL and DMSO were comparable to each other but significantly lower than those in DMF (Figure 3a). Precluding catalytic activity of the solvents as an explanation to these differences (Supporting Information Figure 9), leaves differences in solvation of Sn(Oct)_2_ as a likely explanation to the different rates of depolymerization. This was, however, not further evaluated here. Interestingly, the solvent also had a large effect on the epimerization and diastereomeric purity (Figure 3b). The amount of *meso*‐LA in the generated LLA was more than 10‐fold higher in DMSO than in DMF as the reaction reached equilibrium (>95 % conversion to monomer). This result might be explained by differences in basicity, where DMSO is a stronger Lewis base than DMF (donor numbers: DMSO=29.8, DMF=26.6),^[36]^ and epimerization is a consequence of base‐promoted α‐proton abstraction.^[37]^ For the same reason, the fraction of *meso*‐LA in the crude LLA increased to 20 % and 30 % as Sn(Oct)_2_ was exchanged for the organic base catalysts 1,5,7‐triazabicyclo[4.4.0]dec‐5‐ene (TBD) and 1,8‐diazabicyclo(5.4.0)undec‐7‐ene (DBU) (Supporting Information Tables 2.9–2.14). In addition to epimerization, concern has been raised about cis‐elimination as an obstructing side reaction in the CRM of PLLA.[^1d^, ^25^] This reaction would result in the formation of acrylic acid and other acrylic oligomers,[^24^, ^38^] thereby reducing both the yield and purity of the generated LLA. This reaction was not observed in either DMF or GVL, but traces of acrylic acid, approximately 0.1 % of all converted PLLA, could be identified in DMSO after 1 h at 140 °C. This side reaction turned out to be accelerated both by increased temperature and by dilution, where, for example, the acrylic acid content increased to 20 % of all converted PLLA after 2 h at 180 °C with an initial PLLA concentration of 0.25 M (see Supporting Information Tables 2.15–2.18). Hence, compared with DMSO, both DMF and GVL gave a higher selectivity determined as the proportion of LLA in the crude lactide mixture (Equation (SE2) in Supporting Information Note 1) toward LLA. However, all solvents exhibited high conversions >95 % (DMF: 99 % selectivity at 96 % conversion; GVL: 98 % selectivity at 96 % conversion; DMSO: 92 % selectivity at 97 % conversion).

Due to the large interest in recycling mixed plastic waste streams, we evaluated whether PLLA could be depolymerized in the presence of common packaging plastics used in similar applications as PLA. Pieces of three different postconsumer plastics composed of polyethylene (PE), polypropylene (PP) and poly(ethylene terephthalate) (PET) (Supporting Information Figure 10) were added to a solution of PLLA (*M*_n_=14 000 g mol^−1^, *Ð*=1.13) and DMF (0.5 M PLLA based on the repeating unit of LLA), together with 10 mol % Sn(Oct)_2_. The depolymerization was carried out at 140 °C and, although the presence of mixed plastic waste appeared to slow down the reaction and slightly lowered the polymer‐to‐monomer conversion (1 h: 90 %; 1.5 h: 93 %) compared with that after depolymerization of PLLA alone under the same conditions (1 h: 96 %; 2 h: 98 %), the selectivity remained high (98 % selectivity at 93 % conversion; Supporting Information Table 1.19). All PE, PP and PET pieces remained intact as one piece (Figure 3c) and did not show signals in ^1^H‐NMR. Extending the mixed waste to also include PA6.6, and replacing in lab synthesized PLLA to pieces cut from a PLA cup (Supporting Information Figure 10) slowed down the reaction further (3 h: 86 % conversion), even though no degradation products from PA6.6. were observed in ^1^H‐NMR. Including PC to the mixed waste inhibited the depolymerization (2 h: 13 % conversion), where PC dissolved in DMF and changes in the ^1^H‐NMR aromatic region indicated formation of bis‐phenol A and transesterification with PLA. To conclude, the presence of several common plastics (PE, PP, PET and PA6.6) only decrease the depolymerization rate, but the depolymerization stays selective, targeting PLA in a mixed plastic waste stream.

We have now successfully demonstrated how PLLA can be depolymerized to LLA by ring‐closing depolymerization in either DMF or GVL. However, is this only an effect of dilution, or are there other factors involved? According to the Dainton–Ivin equation 1,^[2a]^

(1) $$\frac{\Delta H_{p}^{o}}{RT}-\frac{\Delta S_{p}^{o}}{R}=\ln{\left[M\right]}_{eq}$$

the equilibrium monomer concentration [M]_eq_ for equilibrium chain‐growth polymerization depends on Δ*H*_p_, Δ*S*_p_ and temperature *T*. In a system where both Δ*H*_p_ and Δ*S*_p_ are negative, [M]_eq_ increases with increasing reaction temperature. Eventually, a critical temperature is reached, above which polymerization is thermodynamically forbidden. This temperature is referred to as the ceiling temperature *T*_c_ and is defined as the temperature at which [M]_eq_=[M]_0_. Consequently, *T*_c_ is dependent on the initial monomer concentration [M]_0_, which is why *T*_c_ naturally decreases as polymerization is performed in solution compared to bulk. From the Dainton–Ivin equation (1), one may erroneously conclude that the equilibrium behavior is independent of the solvent and that only the temperature and [M]_0_ matter. However, often forgotten is that the equation assumes all monomer–polymer–solvent interactions to be negligible.^[3]^ In the case of polymerization of heterocyclic monomers, there are several examples in the literature where this assumption clearly does not hold and in which the properties of the solvent influence the equilibrium behavior.[^31^, ^32^] Hence, we believed that the depolymerization of PLA to LA presented in this work could possibly be attributed to monomer–polymer–solvent interactions as an additional factor to the effect of dilution.

**Figure 4:**
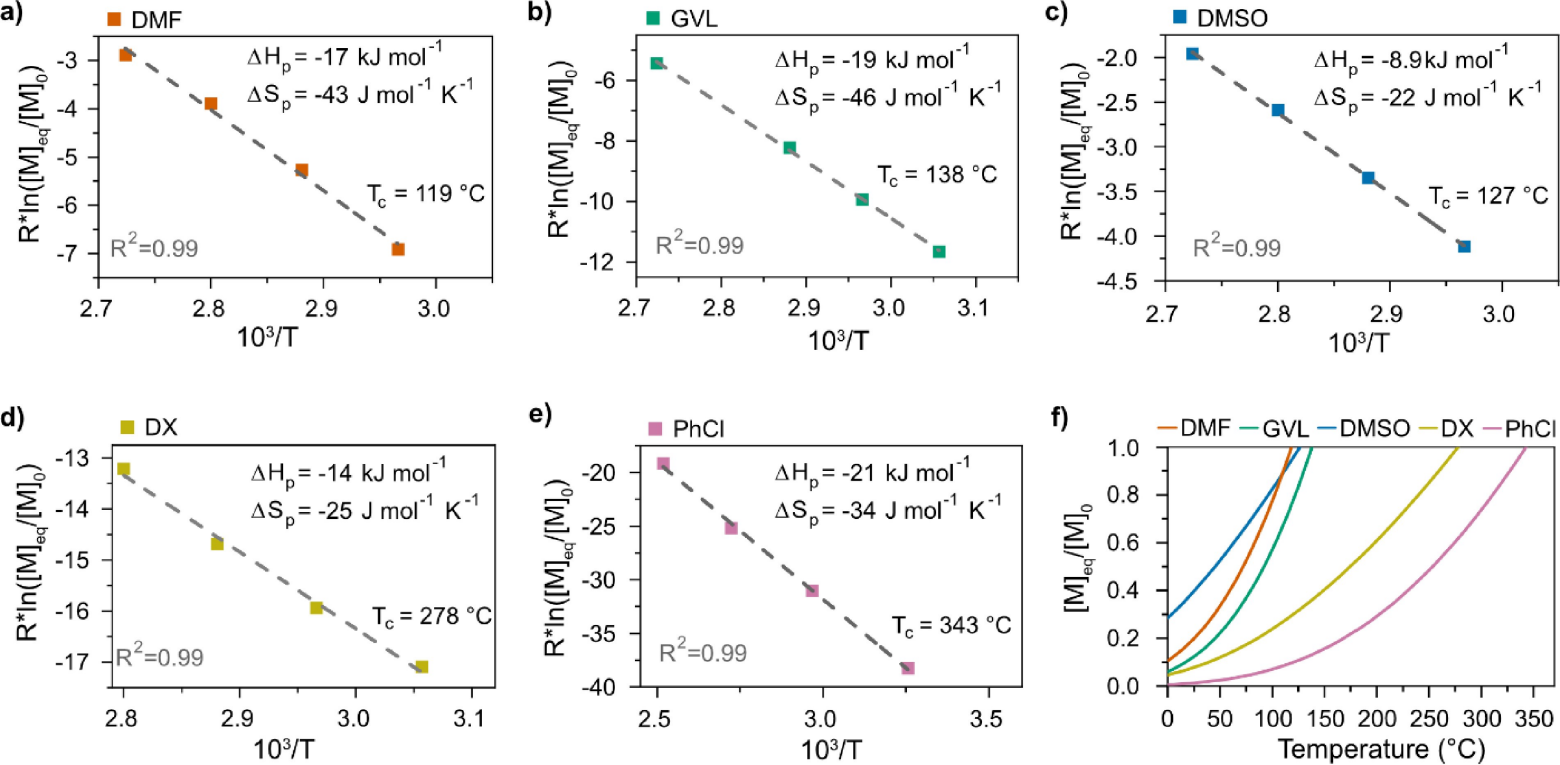
Polymerization thermodynamics of LLA. Initial monomer concentration: [M]_0_=0.5 M. Catalyst (DBU) concentration: 10 mol %. a) Calculations of Δ*H*_p_, Δ*S*_p_ and *T*_c_ of LLA in DMF. b) Calculations of Δ*H*_p_, Δ*S*_p_ and *T*_c_ of LLA in GVL. c) Calculations of Δ*H*_p_, Δ*S*_p_ and *T*_c_ of LLA in DMSO. d) Calculations of Δ*H*_p_, Δ*S*_p_ and *T*_c_ of LLA in DX. e) Calculations of Δ*H*_p_, Δ*S*_p_ and *T*_c_ of LLA in PhCl. f) Theoretical equilibrium behavior ([M]_eq_/[M]_0_) as a function of temperature.

**Figure 5:**
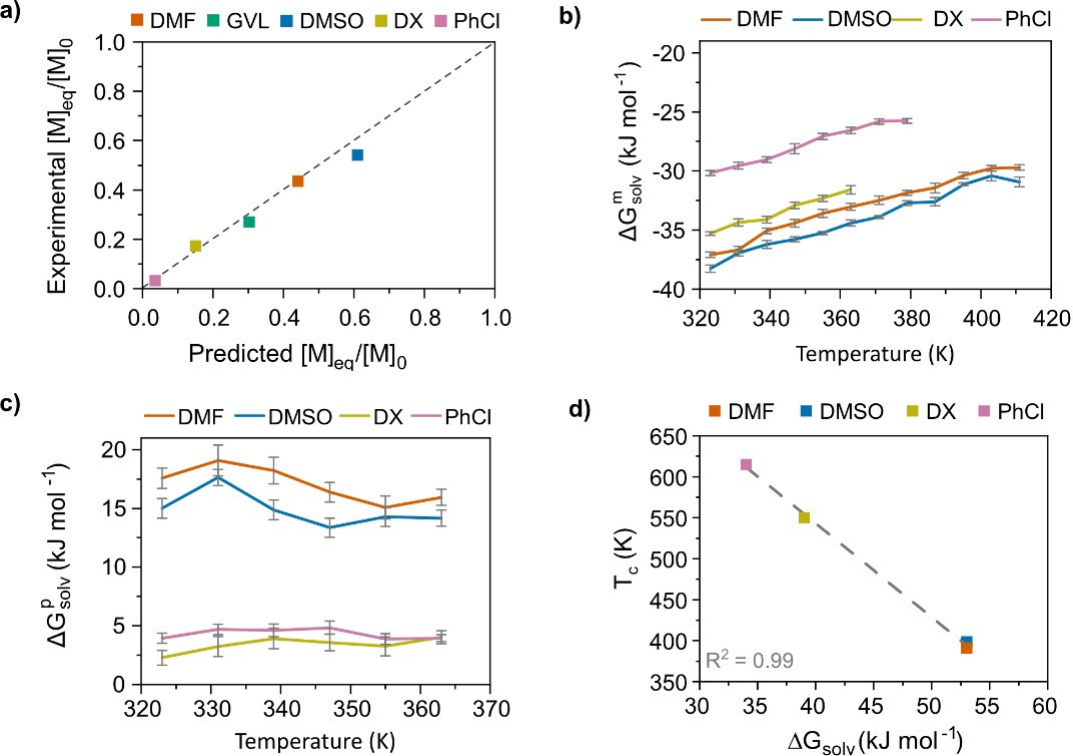
Relationship between depolymerization behavior and thermodynamics of polymerization and solvation. a) The experimental monomer concentration at equilibrium [M]_eq_/[M]_0_ from depolymerization at 65 °C ([M]_0_=0.5 M) in relation to the equilibrium monomer concentration predicted from Δ*H*_p_ and Δ*S*_p_ in each solvent. b) The free energy of solvation of LLA (Δ*G* ^m^_solv_) in relation to temperature, calculated through MD simulations of LLA in DMF, DMSO, DX and PhCl. Δ*S* ^m^_solv_ is determined from the Δ*G* ^m^_solv_‐temperature slope. c) The free energy of solvation of PLLA (Δ*G* ^p^_solv_) in relation to temperature, calculated through MD simulations of PLLA in DMF, DMSO, DX and PhCl. Δ*S* ^p^_solv_ is determined from the Δ*G* ^p^_solv_‐temperature slope. d) The experimentally calculated *T*_c_ as a function of Δ*G*_solv_=Δ*G* ^p^_solv_−Δ*G* ^m^_solv_ at 323 K (Δ*G* ^p^_solv_ and Δ*G* ^m^_solv_ calculated from MD simulations).

The polymerization thermodynamics (Δ*H*_p_, Δ*S*_p_ and *T*_c_) of LLA at [M]_0_=0.5 M were, therefore, determined in five different solvents: DMF, GVL, DMSO, 1,4‐dioxane (DX) and chlorobenzene (PhCl) (Figure 4; Supporting Information Table 3 and Supporting Information Note 2). Note that the calculated *T*_c_ values in DMF, GVL and DMSO are all below 140 °C (the lowest reaction temperature in the depolymerization experiments presented in Figure 2 and 3), explaining why a polymer conversion >95 % could be reached in all three solvents. In addition, the difference between the lowest and highest *T*_c_ (DMF: *T*_c_=119 °C; PhCl: *T*_c_=343 °C) is significant and verifies that *T*_c_ is considerably affected by the properties of the solvent and not only by the concentration. Moreover, equilibrium depolymerization at 65 °C resulted in a molar fraction [M]_eq_/[M]_0_ which clearly agreed with values predicted from the thermodynamic data (Figure 5a, Supporting Information Table 4). This result supports the concept that the depolymerization of PLA is related to the polymerization thermodynamic parameters of LA and, consequently, the formation of LLA as a depolymerization product.

However, the actual role of the solvent and controlling factors behind the differences in the *T*_c_ depression are still unclear. To elucidate how the solvent properties correlate to the *T*_c_ depression, we turned to theories of solubility. Based on Flory–Huggins theory, Ivin and Léonard^[29]^ have shown how interactions between monomer, polymer and solvent affect the monomer–polymer equilibrium through the interaction parameters between monomer–solvent (*χ*_ms_), solvent–polymer (*χ*_sp_) and monomer–polymer (*χ*_mp_):

(2) $$\frac{{\Delta H}_{p}}{RT}-\frac{{\Delta S}_{p}}{R}=ln\varphi_{m}+1+\left(\chi_{ms}-\chi_{sp}\left(\frac{V_{m}}{V_{s}}\right)\right)\varphi_{s}+\chi_{mp}\left(\varphi_{p}-\varphi_{m}\right)$$

Here φ denotes the mole fractions and V the molar volumes of solvent (s), monomer (m) and polymer (p). The Flory–Huggins interaction parameter can be understood as the deviation from the ideal free energy of mixing originating in monomer‐solvent interactions, which includes both enthalpic and entropic contributions. *χ* has a very direct relationship with the excess free energy of mixing Δ*G*_solv_ and, hence, one can estimate interaction parameters by atomistic molecular dynamics simulations, which give Δ*G*_solv_ directly. Here, Δ*G*_solv_ was calculated for both the monomer (LLA) and polymer (oligomer, oPLLA) in four different solvents as a function of temperature (Figure 5b, c). The Δ*G*_solv_ of LLA (Δ*G* ^m^_solv_) increased with temperature in all solvents. Interestingly, although the magnitude is different, the slope of Δ*G* ^m^_solv_ as a function of T is the same in all cases, which means that the entropy of solvation for the monomer is independent of the solvent and that the enthalpy is independent of T. In addition, the Δ*G*_solv_ of oPLLA (Δ*G* ^p^_solv_) shows that the entropy of solvation for the polymer is small compared to the enthalpy. This finding leads to the important conclusion that Δ*S*_p_ in Equation (2) is independent of the solvent used, which in turn leads to the variation in *T*_c_ among the various solvents being linear with Δ*H*_p_ in Equation (1). Indeed, when the experimentally determined *T*_c_ values are plotted as a function of the calculated total Δ*G*_solv_=Δ*G* ^p^_solv_−Δ*G* ^m^_solv_ (Supporting Information Note 4), they fall on a straight line (Figure 5d). Therefore, we continued to investigate whether the Hildebrand solubility parameter could be used to approximate the *T*_c_ depression in different solvents.

The Hildebrand solubility parameter (*δ*) can be calculated from physical data which can be found in literature and is readily available for a broad range of solvents. In line with the principle of “like dissolves like”, the closer a solvent and solute are in solubility parameter or the smaller, e.g., the term (*δ*_m_−*δ*_s_)^2^ is, the higher is the expected miscibility between the two compounds.^[39]^ In contrast to *χ*, which contains both enthalpic and entropic terms, δ only contains the enthalpic contribution. However, since MD simulations indicated that the entropy term was negligible, they are related to the interaction parameters through:

(3) $$\chi_{ms}=\frac{V_{m}{\left(\delta_{m}{-\delta }_{s}\right)}^{2}}{RT}$$

(4) $$\chi_{ps}=\frac{V_{p}{\left(\delta_{p}-\delta_{s}\right)}^{2}}{RT}$$

**Figure 6:**
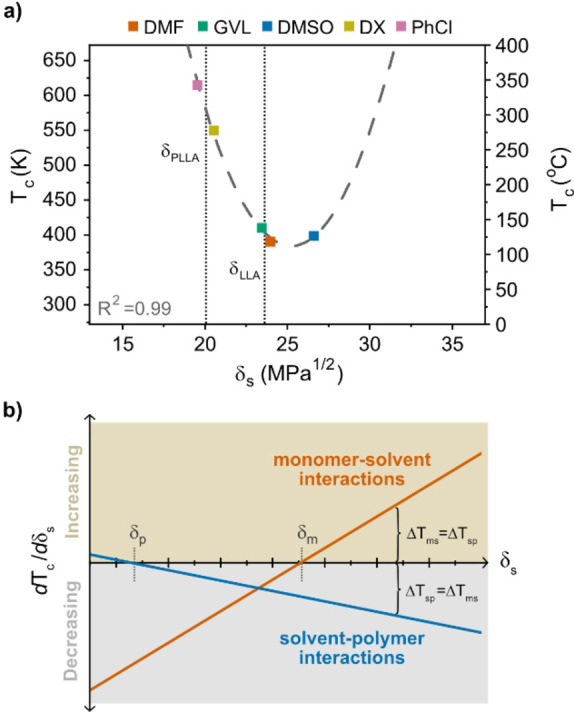
Ceiling temperature (*T*_c_) of LLA in relation to *δ*_s_. a) *T*_c_ determined from Δ*H*_p_ and Δ*S*_p_ in each solvent (Figure 4a–e). Initial monomer concentration: [M]_0_=0.5 M. Catalyst (DBU) concentration: 10 mol %. b) The change in *T*_c_ with increasing *δ*_s_ (d*T*_c_/d*δ*_s_) due to monomer–solvent interactions (orange) and solvent–polymer interactions (blue). The color of the area indicates whether the effect is positive/increasing (beige) or negative/decreasing (gray). The highest *T*_c_ depression is obtained at *δ*_s_, where Δ*T*_ms_=Δ*T*_ps_.

As a result, the variation in ceiling temperature among different solvents characterized by the Hildebrand solubility parameters *δ*_s_ can be written as:

(5) $$T_{c}=a{\left(\delta_{m}-\delta_{s}\right)}^{2}+b{\left(\delta_{s}-\delta_{p}\right)}^{2}+c$$

where *a*, *b*, and *c* are parameters that can be determined by fitting. In Figure 6a, it is shown that using literature values for the solubility parameters (Supporting Information Table 5), this function fits extremely well with the experimental *T*_c_ (R^2^=0.99). From multiple linear regression, applied to determine the coefficients *a*, *b* and *c* in Equation (5) (*a*=10.8 K MPa^−1^, *b*=−3.53 K MPa^−1^, *c*=444 K; R^2^=0.99; Supporting Information Table 6), we concluded that the monomer‐solvent interactions, (*δ*_m_−*δ*_s_)^2^, appeared to have the largest influence on *T*_c_, which was increased by 11 K per unit MPa. Hence, this means that a low *T*_c_ is promoted by a solvent with *δ*_s_ close to *δ*_m_, which conforms with Ivin‐Léonard theory. However, the optimum *δ*_s_ does not simply equal *δ*_m_, as the correlation between the solvent‐polymer interactions, (*δ*_s_−*δ*_p_)^2^, and *T*_c_ is negative, having a decreasing effect on *T*_c_ by 3.5 K per MPa.

It may be tempting to assume that the *T*_c_ of LLA could be further decreased by increasing the polarity of the solvent. However, there appears to be a limit of the extent to which *T*_c_ can be depressed by tuning the solvent properties. As illustrated in Figure 6b, *T*_c_ is influenced by both monomer‐solvent interactions and solvent‐polymer interactions. The change in *T*_c_ with increasing *δ*_s_ (d*T*_c_/d*δ*_s_), due to solvent‐polymer interactions, is positive for *δ*_s_<*δ*_p_; however, as *δ*_s_ exceeds *δ*_p,_ the effect becomes negative. Hence, if only solvent‐polymer interactions were operative, *T*_c_ would continue to decrease with increasing *δ*_s_. However, the effect of the solvent‐monomer interactions is stronger and turns from negative to positive as *δ*_s_ exceeds *δ*_m_. Therefore, as the decreasing effect from solvent‐polymer interactions is outpaced by the increasing effect from monomer‐solvent interactions (Δ*T*_ms_=−Δ*T*_sp_), *T*_c_ reaches its minimum.

## Conclusion

The efficient and selective depolymerization of PLLA into the cyclic monomer LLA has been considered a significant challenge due to the high *T*_c_ of LLA and the accompanying epimerization and elimination reactions. This is why CRM of PLLA has often been ruled out as a possibility.[^1d^, ^25^] Nevertheless, herein, we have demonstrated how PLLA can be chemically recycled to a yield >95 %, with 98–99 % selectivity, within 1–4 h at 140 °C. This process was enabled through solution depolymerization in either DMF (99 % selectivity at 96 % conversion) or the green solvent GVL (98 % selectivity at 96 % conversion) at 0.5 M. In this process, the *T*_c_ of LLA could be decreased to 119 °C (DMF) or 138 °C (GVL). To ensure selective depolymerization to LLA with high diastereomeric purity, the choice of both solvent and catalyst appeared essential in suppressing epimerization and elimination reactions. In addition, depolymerization selectively targeted PLLA in a mixed plastic waste stream.

Apart from influencing the selectivity of the reaction, the solvent properties, in terms of monomer–polymer–solvent interactions, were the key to successful *T*_c_ depression. Hence, the easily obtained δ can be employed to screen and predict which solvent should be the most suitable to promote ring closing, following a “like recycles like” principle.

Knowledge of how monomer–polymer–solvent interactions promote *T*_c_ depression is powerful information toward selective chemical recycling. Although previous studies have shown that solvent interactions influence monomer–polymer equilibrium, their potential by means of chemical recycling remains to be explored.[^29^, ^30^, ^31b^, ^32^] With this study, we have taken the first step on this exciting journey and, although the equilibrium between PLA and LA was the focus of this study, we believe this behavior can be translated to other monomer‐polymer systems as well. The system can potentially close the loop of PLA on a large scale and, more importantly, give inspiration on how to design a future fully circular material economy.

## Conflict of interest

The authors declare no conflict of interest.

1

## Supporting information

As a service to our authors and readers, this journal provides supporting information supplied by the authors. Such materials are peer reviewed and may be re‐organized for online delivery, but are not copy‐edited or typeset. Technical support issues arising from supporting information (other than missing files) should be addressed to the authors.

Supporting Information

## Data Availability

The data that support the findings of this study are available from the corresponding author upon reasonable request.
