# Supplementary material for: “Like Recycles Like”: Selective Ring‐Closing Depolymerization of Poly(L‐Lactic Acid) to L‐Lactide
Source: Angew Chem Int Ed Engl. 2022 Jun 9;61(33):e202204531. doi: 10.1002/anie.202204531 (PMC9541399; doi:10.1002/anie.202204531)
Supplement: Supplementary file 1 — Supporting Information [file ANIE-61-0-s001.pdf]

Angewandte Chemie
Eine Zeitschrift der Gesellschaft Deutscher Chemiker

Supporting Information

“Like Recycles Like”: Selective Ring-Closing Depolymerization of Poly(L-Lactic Acid) to L-Lactide

*L. Cederholm, J. Wohlert, P. Olsén, M. Hakkarainen, K. Odelius**

Supporting Information

Table of Contents

Experimental section S2

Materials S2

Polymerization of LLA S2

Depolymerization of PLA S3

Repolymerization of LA S3

Characterization S4

Molecular dynamics (MD) simulations S4

Synthesis and characterization of PLA S5

Supporting Tab. 1. Synthesis of PLLA. S5

Supporting Fig. 1. Molecular weight data. S5

Supporting Fig. 2. PLLA and PLA used for depolymerization. S6

Depolymerization of PLLA S6

Supporting Fig. 3. DMF stability at 140 °C in the presence of $Sn(Oct)_2$. S6

Supporting Fig. 4. 1H NMR peak assignment. S7

Supporting Note 1. Calculation of relative amounts of LLA, DLA meso-LLA and PLLA. S7

Supporting Tab. 2. Depolymerization kinetics. S8

Supporting Fig. 5. Decrease in molecular weight in relation to polymer conversion. S11

Supporting Fig. 7. Direct repolymerization. S12

Supporting Fig. 8. Isolation of recycled LA. S13

Supporting Fig. 9. Catalytic activity of solvents. S14

Supporting Fig. 10. PLA recycling in the presence of mixed plastic waste. S14

Polymerization thermodynamics S15

Supporting Tab. 3. Data for determination of polymerization thermodynamic parameters. S15

Supporting Note 2. Calculation of polymerization thermodynamic parameters. S15

Supporting Note 3. Polymerization thermodynamics for short linear chains. S16

Supporting Tab. 4. Relationship between depolymerization behaviour and polymerization thermodynamics. S16

Relationship between solubility parameters and T_c S17

Supporting Note 4. Variation in T_c with different solvents. S17

Supporting Tab. 5. Hansen's solubility parameters. S18

Supporting Tab. 6. Relationship between solubility parameters and T_c. S19

Molecular modelling S19

Supporting Fig. 11. Liquid densities as a function of temperature. S19

Supporting references S20

Experimental section

Materials

L-Lactide (LLA, Boehringer-Ingelheim), purified through recrystallization in toluene (Fischer Scientific, > 99.8%), was used to synthesize poly(L-lactic acid) (PLLA), and all polymerizations were performed with benzyl alcohol (BnOH, Sigma–Aldrich, anhydrous, 99.8%, stored over molecular sieves) as the initiator. Tin(II) 2-ethylhexanoate ($Sn(Oct)_2$, Sigma–Aldrich, 92.5-100%, stored over molecular sieves), 1,8-diazabicyclo[5.4.0]undec-7-ene (DBU, Sigma–Aldrich, > 99.0%) and 1,5,7-triazabicyclodec-5-ene (TBD, Sigma–Aldrich, 98%) were used as catalysts for polymerization and/or depolymerization reactions. *N,N*-Dimethylformamide (DMF, Sigma–Aldrich, anhydrous, 99.8%), γ-valerolactone (GVL, Sigma–Aldrich, ReagentPlus, 99%, stored over molecular sieves), dimethyl sulfoxide (DMSO, VWR Chemicals, anhydrous), acetone (VWR Chemicals, technical grade, > 99%, stored over molecular sieves), toluene (PhMe, Sigma–Aldrich, anhydrous, 99.8%), chloroform ($CHCl_3$, Fisher Scientific, analytical reagent grade, > 99.8%, stored over molecular sieves), 1,4-dioxane (DX, Sigma–Aldrich, anhydrous, 99.8%) and chlorobenzene (PhCl, Fluka Chemika, > 98%, stored over molecular sieves) were used as solvents in the depolymerization experiments. For the end capping experiment, acetyl chloride (Acros Organics, >99%), triethylamine (Sigma Aldrich, >99.5%) and dichloromethane (CH_2Cl_2, Sigma Aldrich, anhydrous, >99.8%) were used without further purification. Chloroform ($CHCl_3$, Fisher Scientific, analytical reagent grade, > 99.8%), heptane (VWR Chemicals, 99.8%) and methanol (VWR Chemicals, technical grade, > 98.5%) were used for purification of synthesized PLLA through precipitation. For all NMR analyses, deuterated chloroform ($CDCl_3$, VWR Chemicals, 99.8%) was used as the solvent, and $CHCl_3$ was used as the internal reference. Poly(lactic acid) (PLA) Ingeo^{TM} Biopolymer 4043D (NatureWorks, DLA content: 3-5%) was included as commercial grade PLA. The PLA arrived as granules, which were grinded to finer particles during cooling by liquid nitrogen (Supporting Fig. 2) prior to depolymerization. Pieces of postconsumer plastic of PLA, polyethylene (PE), polypropylene (PP), poly(ethylene terephthalate) (PET) and polycarbonate (PC) (Supporting Fig. 10) and pellets of polyamide 6.6 (PA6.6, Sigma Aldrich) represented a mixed plastic waste stream.

Polymerization of LLA

Synthesis of PLLA

PLLA was synthesized in bulk by varying the ratio of initiator (BnOH) to monomer (LLA) to target different molecular weights (Supporting Table 1.1-.1.4). As an example of the general procedure, LLA (20.0 g, 0.139 mol) was added to a round-bottom flask together with BnOH (0.150 g, 0.00139 mol) and $Sn(Oct)_2$ (0.563 g, 0.00139 mol) as catalysts. ROP was carried out under a N_2 atmosphere at 100 °C for 1 h, after which the reaction was quenched by cooling. The polymer was dissolved in $CHCl_3$ and precipitated in a mixture of cold heptane and methanol (10 mol% methanol). The precipitation was repeated four times to remove $Sn(Oct)_2$ and unreacted monomers. The purified polymer was dried in a fume hood overnight and thereafter under vacuum (4 days at room temperature and 2 days at 60 °C). The polymer was stored in a glove box under a N_2 atmosphere.

End capping of PLLA

PLLA ($M_{n,SEC}$ = 14.1 kDa, Đ = 1.13; Supporting Table 1.1) (1.0 g, 0.42 mmol -OH end groups) was added to a 25 mL round bottom flask equipped with a magnetic stirrer together with CH_2Cl_2 (10 mL) and triethylamine (290 μL, 2.1 mmol). Acetyl chloride (75 μL, 1.1 mmol) was added dropwise under stirring. The flask was kept under a N_2 atmosphere, and the reaction was let to proceed for 1 h at RT (Supporting Figure 6). The solution was washed with HCl_{aq} (10 mL, 0.5 M) followed by water (10 mL) and brine (3x10 mL). CH_2Cl_2 was removed on the rotary evaporator followed by drying under vacuum for 2 days.

Calculation of polymerization thermodynamic parameters
To calculate the thermodynamic parameters (ΔH_p and ΔS_p) of LLA polymerization in different solvents (DMF, GVL, DMSO and PhCl), polymerization was carried out in solution ($[M]_0$ = 0.5 M LLA) at four different temperatures per solvent. As a general procedure, LLA (0.144 g, 1.00 mmol) was added to a 10 mL flask equipped with a magnetic stirrer together with BnOH (0.1 mL of 1 mM DMF stock solution, 0.01 mmol) and 1.7 mL of DMF (Supporting Table 3). The preparation of the reaction vessel was performed inside a glove box under a N_2 atmosphere. The vial was sealed with an aluminum/Teflon crimp cap with a rubber septum before being transferred out of the glove box into the open laboratory. The vial was immersed in a preheated oil bath, and the reaction was started by adding DBU (0.2 mL of 0.5 M DMF stock solution, 0.1 mmol) through the septum with a syringe. Aliquots of 0.1 mL were removed through the septum at regular time intervals, quenched by cooling in 0.6 mL of $CDCl_3$, and analyzed by ^{1}H NMR spectroscopy. The reaction was allowed to proceed until an equilibrium monomer concentration was established. This process was repeated at four different temperatures, and the equilibrium monomer concentration $[M]_{eq}$ was recorded. Thus, ΔH_p and ΔS_p were determined via linear regression of $R*ln([M]_{eq}/[M]_0)$ as a function of $1/T$ (Supporting Note 2).

Depolymerization of PLA

Six different polymers with varying molecular weights were utilized for depolymerization experiments. Four polymers were synthesized as described above, while the fifth was commercial grade PLA (PLA Ingeo^{TM} Biopolymer 4043D, NatureWorks) (Supporting Fig. 1). The fifth polymer was end capped as described above. All polymers were used as crushed powders (Supporting Fig. 2).

The solvent, polymer concentration, catalyst, catalyst concentration and temperature were varied to explore the effect on the depolymerization and side reactions. In general, PLLA (0.072 g or 0.144 g; 0.5 or 1.0 mmol) was added to a 10 mL flask equipped with a magnetic stirrer together with catalyst (1-10 mol %; $Sn(Oct)_2$, DBU or TBD) and 2.0 mL of solvent (DMF, GVL, DMSO, PhCl, DX, acetone, $CHCl_3$ or PhMe), resulting in a concentration of 0.5 M (based on the LLA repeating unit). The preparation was performed inside a glove box under a N_2 atmosphere. The vial was sealed with an aluminum/Teflon crimp cap with a rubber septum before being transferred out of the glove box. The vial was quickly heated with a heating gun to obtain a homogenous solution directly before it was immersed in a thermostatic oil bath (65 °C, 140 °C 160 °C or 180 °C). Aliquots of 0.1 mL were removed through the septum at regular time intervals, quenched by cooling in 0.6 mL of $CDCl_3$, and analysed by ^{1}H NMR spectroscopy without further purification.

For depolymerization in a mixed plastic waste stream, pieces of postconsumer plastic composed of PE, PP, PET and PC (Supporting Fig. 10) and pellets of PA6.6 were used. The experiments were performed with both PLLA (M_n = 14,000 g mol^{-1}, Đ = 1.13) and with pieces cut from a PLA cup (Supporting Figure 10). The mixed plastics were added to DMF (0.5 PLLA/PLA based on the LL/LA repeating unit). After depolymerization the insoluble plastic pieces were separated from the solution by filtration. The pieces were photographed before and after the reaction (**Figure 3c**, Supporting Figure 10).

Repolymerization of LA

Direct repolymerization
PLLA ($M_{n,SEC}$ = 14.1 kDa, Đ = 1.13; Supporting Table 1.1) was depolymerized in DMF (0.5 M based on the LLA repeating unit) at 140 °C (details described above). 10 mol% TBD was added directly to the reaction mixture. The polymerization was performed at RT, and aliquots were withdrawn at regular time intervals, quenched by acetic acid, and analyzed by ^{1}H NMR spectroscopy without further purification.

Isolation and repolymerization of LA
PLA (3.60 g, 25.0 mmol: Ingeo^{TM} Biopolymer 4043D, NatureWorks, M_n = 110 kDa, Đ = 2.02; Supporting Figure 1) was depolymerized in DMF (50 mL) with $Sn(Oct)_2$ (1.01g, 2.50 mmol) at 140 °C. The sample was

concentrated from 0.5 M to 3 M LLA by distillation of DMF at 25 °C under dynamic vacuum (0.2-0.3 mbar) for 10 h. Thereafter, 50 mL of *n*-heptane was added and distilled off at 25 °C. This was repeated four times until a thick viscous slurry was obtained. LA was recrystallized from the slurry in toluene, and the crystals were dried under vacuum overnight. ROP of recycled LA was performed as described above (details in Supporting Table 1.5). The crude polymer was analysed by ^{1}H NMR and SEC without further purification (Supporting Figure 1).

Characterization

Nuclear magnetic resonance (NMR) spectroscopy

^{1}H NMR spectra were recorded on a Bruker Avance III HD (400 MHz) spectrometer. All experiments were performed at RT with $CDCl_3$ as the solvent and with $CHCl_3$ as the internal reference.

Size exclusion chromatography (SEC)

Molecular weight analysis via SEC was performed on a Malvern GPCMAX instrument equipped with an autosampler, a PLgel 5 µm guard column (7.5 x 50 mm) and two PLgel 5 µm MIXED-D (300 x 7.5 mm) columns. The polymer sample (4-5 mg mL^{-1}) was dissolved in chloroform containing 2% v/v toluene, which also was used as eluent. The flow rate was 0.5 mL min^{-1}, and the temperature was kept at 35 °C. Narrow disperse polystyrene standards with molecular weights in the range of 1,200-400,000 g mol^{-1} were used for calibration.

Molecular dynamics (MD) simulations

All MD simulations were performed using GROMACS[1] version 2020.2 using a stochastic integration algorithm[2] with a basic time step of 2 fs. Nonbonded interactions were cut off at 1.2 nm and shifted to ensure zero potential at the boundary. Electrostatic interactions were treated with PME[3,4] using a real-space cutoff of 1.2 nm. Pressure was maintained at 1 atm using a Parrinello-Rahman barostat[5] and a compressibility of $5 \cdot 10^{-5}$ bar^{-1}, while temperature was controlled by the integration algorithm. All covalent bonds were constrained to their equilibrium value using P-LINCS.[6] All simulations employed a replica exchange protocol[7] using eight temperatures ranging from 323 K to 363 K in steps of 8 K. Exchange between neighboring replicas was attempted every 1000 steps.

The simulations were run on cyclic L-lactide and PLLA oligomers of DP 5 or 6 in different solvents using a fully periodic computational box in the shape of a truncated octahedron with an approximate nearest-image distance of 3.5 nm. The liquid molecules included in the simulations were DMSO, DMF, DX, and PhCl. All compounds were modeled using the general CHARMM force field (CGenFF) version 2.4.0.[8–10] The liquid densities showed good agreement with the experimental values (Supporting Fig. 11).

Solvation free energies were calculated using Computational Alchemy[11] in which solute-solvent interactions were linearly decoupled using a single coupling parameter. The decoupling was performed in 20 discrete steps, where the first ten were used to decouple electrostatic interactions, followed by van der Waals interactions in the remaining steps. Thus, the fully decoupled state corresponds to the gas phase of the solute and the pure liquid state of the solvent. For each value of the coupling parameter, a 2 ns equilibrium simulation was performed during which the derivative of the total potential with respect to the coupling parameter was sampled. This quantity can finally be connected to the free energy difference between the start and end states, ΔG_S, using Bennett's acceptance ratio.[12]

Synthesis and characterization of PLA

Supporting Tab. 1. Synthesis of PLLA. *Synthesis details and molecular weight analysis.*

	LLA (g) (mmol)	BnOH (g) (mmol)	$Sn(Oct)_2$ (g) (mmol)	Temp. (°C)	Time (h)	Conv. (%)	$M_{n,theo}$[a] (kDa)	$M_{n,NMR}$[b] (kDa)	$M_{n,SEC}$[c] (kDa)	Đ[c]
**1**	20.0<br>139	0.150<br>1.39	0.563<br>1.39	100	1	79	11.4	14.8	14.1	1.13
**2**	50.0<br>347	0.077<br>0.71	0.281<br>0.69	110	15	99	68.5	13.7	7.5	1.73
**3**	1.44<br>10.0	0.055<br>0.51	0.040<br>0.010	100	1	99	2.80	5.35	5.70	1.14
**4**	1.44<br>10.0	0.108<br>1.00	0.041 g<br>0.010	100	1	99	1.43	2.94	2.31	1.21
**5**[d]	0.290<br>2.01	0.002<br>0.02	0.008<br>0.02	100	1	94	13.6	15.4	17.2	1.07

[a]Determined from LLA and BnOH feed and conversion
[b]Determined by end-group analysis of ^{1}H NMR spectra
[c]Determined by $CHCl_3$SEC analysis
[d]Repolymerization of recycled LLA

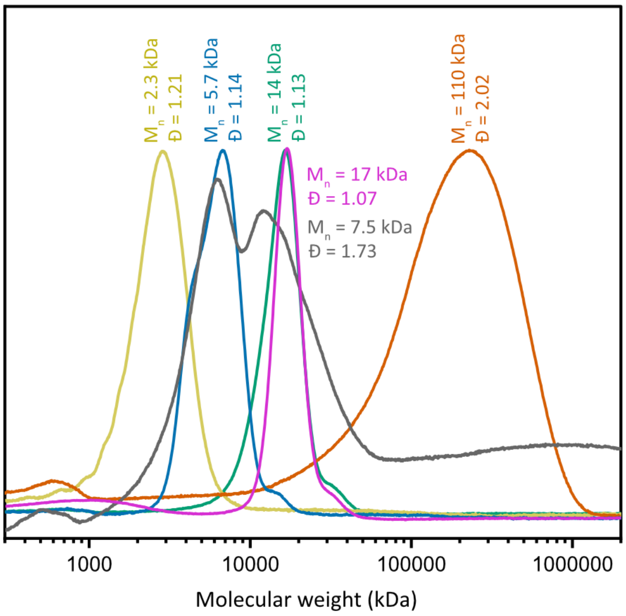

Supporting Fig. 1. Molecular weight data. *SEC molecular weight data of commercial grade PLA (*Ingeo™ Biopolymer 4043D, NatureWorks*) (orange; M_n = 110 kDa, Đ = 2.02), PLLA synthesised in the lab (green, M_n = 14 kDa, Đ = 1.13; grey, M_n = 7.5 kDa, Đ = 1.73; blue, M_n = 5.7 kDa, Đ = 1.14; yellow, M_n = 2.3 kDa, Đ = 1.21) and PLA synthesized from recycled LA (purple, M_n = 17 kDa, Đ = 10.7).*

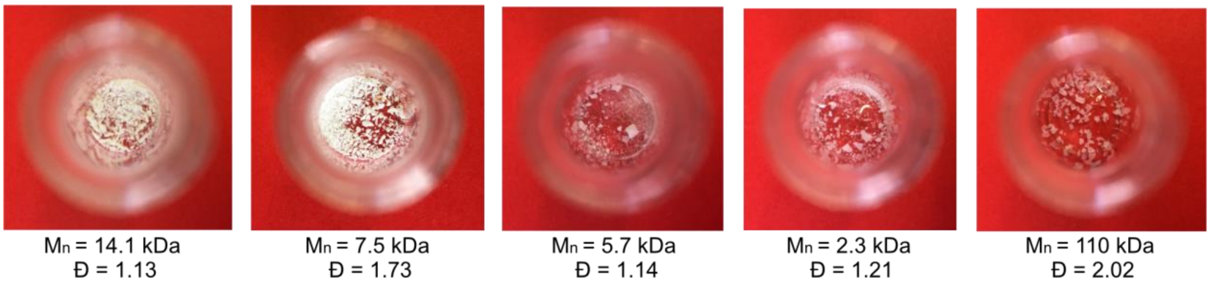

Supporting Fig. 2. PLLA and PLA used for depolymerization. *All images show 10 mg of polymer.*

Depolymerization of PLLA

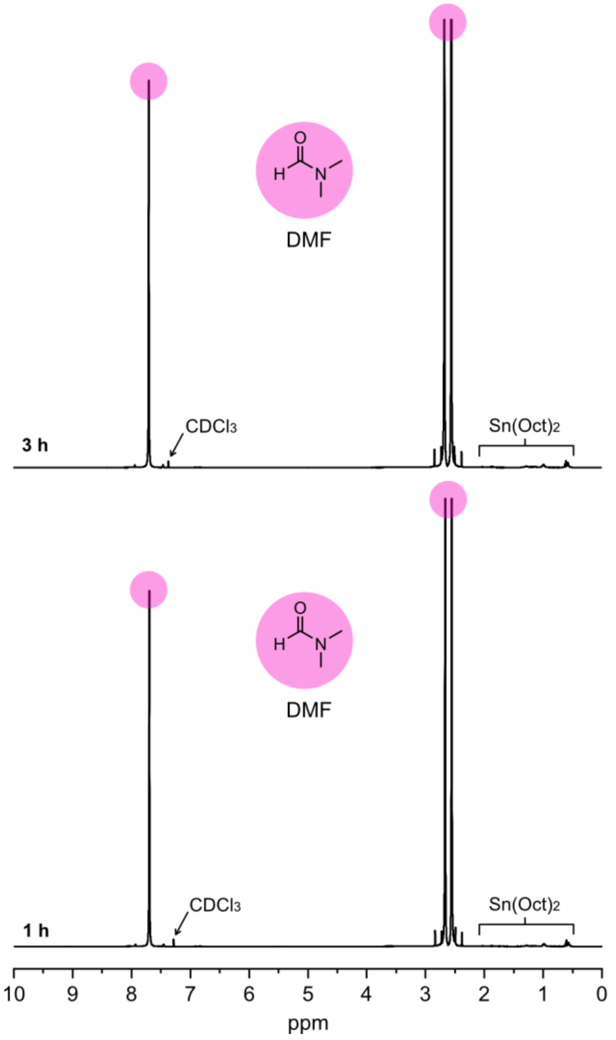

Supporting Fig. 3. DMF stability at 140 °C in the presence of Sn(Oct)$_2$. *Due to concerns regarding potential safety hazards linked to the use of DMF in chemical reactions,*[13] *the stability of DMF under the set reaction conditions was assessed. The figure shows the ^{1}H NMR spectrum of DMF after 1 h and 3 h at 140 °C in the presence of Sn(Oct)$_2$ (0.05 M). No changes could be observed in the spectra within this time range.*

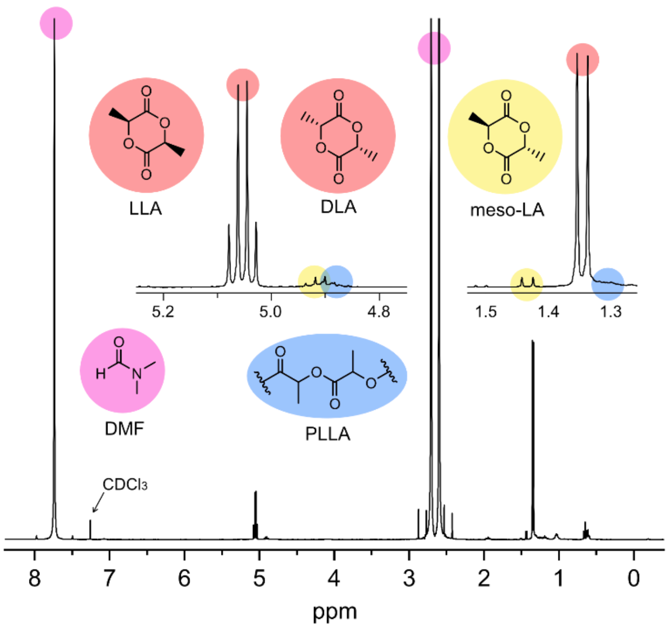

Supporting Fig. 4. 1H NMR peak assignment. *Spectra recorded of sample taken of PLLA (M_n = 14,000, Đ = 1.13) depolymerization in DMF (0.5 M) at 140 °C after 5 h. Due to the presence of DMF in the solution, the chemical shifts are altered as compared to those found in pure $CDCl_3$.*

Supporting Note 1. Calculation of relative amounts of LLA, DLA meso-LLA and PLLA.

*The fraction of meso-LA (b) in the crude lactide mixture was calculated from the integrals of the C**H**$_3$ peaks for meso-LA and LLA/DLA:*

$$b = \frac{\int CH_3(meso-LA)}{\int CH_3(meso-LA)+\int CH_3(LLA/DLA)} \quad \textbf{(SE1)}$$

Based on the probability law, the LLA (L) and DLA (D) content in the crude lactide can be calculated from b accordingly:[14]

$$L = \frac{\left(1+\sqrt{1-2b}\right)^2}{4} \quad \textbf{(SE2)}$$

$$D = \frac{\left(1-\sqrt{1-2b}\right)^2}{4} \quad \textbf{(SE3)}$$

*The total conversion to monomer was calculated from the C**H** peaks for LLA/DLA and meso-LA/PLLA:*

$$Conversion = C = \frac{\frac{\int CH(LLA/DLA)}{L+D}}{\int CH(LLA/DLA)+\int CH(meso-LA/PLLA)} \quad \textbf{(SE4)}$$

Thus, the relative amounts of LLA, DLA, meso-LA, and PLLA presented in Supporting Tab. 1 was calculated accordingly:

$$LLA\ \% = L * C * 100\ \% \quad \textbf{(SE5)}$$

$$DLA\ \% = D * C * 100\ \% \quad \textbf{(SE6)}$$

$$meso - LA = b * C * 100\ \% \quad \textbf{(SE7)}$$

$$PLLA\ \% = (1 - C) * 100\ \% \quad \textbf{(SE8)}$$

Supporting Tab. 2. Depolymerization kinetics. *Depolymerization kinetics and the relative amounts of LLA, DLA, meso-LA, PLA and acrylic acid in DMF, DMSO and GVL. Following parameters were varied: i) PLA and PLLA concentrations; ii) molecular weight; iii) catalyst type iv) catalyst concentration; v) temperature . The DLA content was estimated according to a method based on the law of probability reported elsewhere.*[14]

Reaction	Time (min)	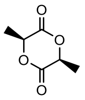 LLA (%)	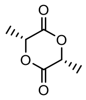 DLA (%)	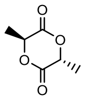 meso-LA (%)	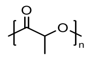 PLA (%)	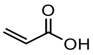 Acrylic acid (%)	Total conv. (%)
**1**							
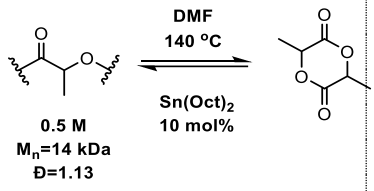	**10**	30.7	< 0.0	< 0.0	69.3	N.D	30.7
	**30**	70.1	< 0.0	0.3	29.6	N.D	70.4
	**60**	96.1	< 0.0	0.7	3.9	N.D	95.5
	**120**	98.4	< 0.0	1.7	1.6	N.D	96.8
	**180**	93.6	< 0.0	2.1	1.4	N.D	91.5
	**240**	94.4	< 0.0	2.8	5.6	N.D	91.5
	**300**	98.8	< 0.0	3.5	1.2	N.D	95.2
**2**							
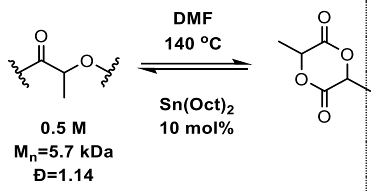	**5**	33.3	< 0.0	< 0.0	66.7	N.D	33.3
	**10**	64.5	< 0.0	< 0.0	35.5	N.D	64.5
	**30**	89.2	< 0.0	0.3	10.8	N.D	89.2
	**60**	87.0	< 0.0	0.7	13.0	N.D	87.0
	**120**	92.4	< 0.0	1.5	7.6	N.D	92.4
	**180**	89.3	< 0.0	2.2	10.7	N.D	89.3
**3**							
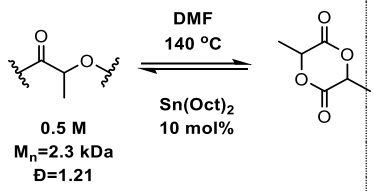	**2**	14.8	< 0.0	< 0.0	85.2	N.D	14.8
	**5**	41.3	< 0.0	< 0.0	58.7	N.D	41.3
	**10**	69.2	< 0.0	0.1	30.8	N.D	69.2
	**30**	84.5	< 0.0	0.4	15.5	N.D	84.5
	**60**	87.6	< 0.0	0.6	12.4	N.D	87.6
	**120**	84.3	< 0.0	1.1	15.7	N.D	84.3
	**180**	81.7	< 0.0	1.7	18.3	N.D	81.7
**4**							
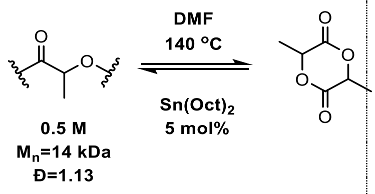	**10**	17.9	< 0.0	0.4	82.1	N.D	17.9
	**30**	52.6	< 0.0	0.2	47.2	N.D	52.8
	**60**	78.5	< 0.0	0.4	21.1	N.D	78.9
	**120**	97.2	< 0.0	0.9	1.9	N.D	98.1
	**180**	93.4	< 0.0	1.4	5.2	N.D	94.8
	**300**	92.4	< 0.0	2.0	5.6	N.D	94.4
	**420**	96.0	< 0.0	3.0	1.0	N.D	99.0
**5**							
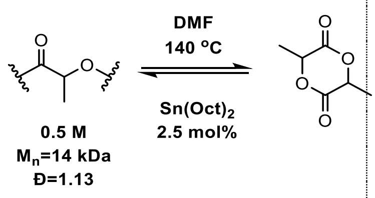	**10**	9.8	< 0.0	< 0.0	90.2	N.D	9.8
	**30**	28.6	< 0.0	< 0.0	71.4	N.D	28.6
	**60**	58.8	< 0.0	0.2	41.2	N.D	58.8
	**120**	88.9	< 0.0	0.7	10.4	N.D	89.6
	**180**	88.9	< 0.0	0.8	10.3	N.D	89.7
	**300**	95.8	< 0.0	1.6	2.5	N.D	97.5
	**420**	95.8	< 0.0	2.2	2.0	N.D	98.0

Reaction	Time (min)	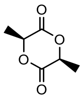 LLA (%)	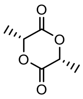 DLA (%)	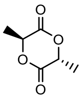 meso-LA (%)	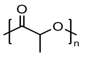 PLA (%)	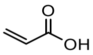 Acrylic acid (%)	Total conv. (%)
**6** 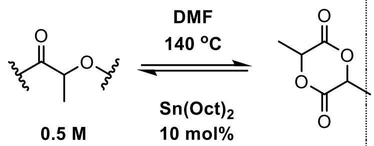 0.5 M Ingeo^{TM} Nature Woks	**10**	23.6*	-	1.4	75.0	N.D	25.0
	**30**	71.9*	-	5.1	23.0	N.D	77.0
	**60**	89.8*	-	6.1	4.2	N.D	95.8
	**120**	94.6*	-	7.5	< 0.0	N.D	> 99.9
	**240**	93.7*	-	8.9	< 0.0	N.D	> 99.9
**7** 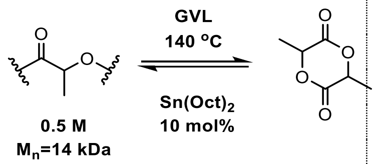 0.5 M M_n=14 kDa Đ=1.13	**10**	19.5	< 0.0	< 0.0	80.5	N.D	19.5
	**30**	57.7	< 0.0	< 0.0	42.3	N.D	57.7
	**60**	79.6	< 0.0	0.3	20.0	N.D	80.0
	**120**	90.8	< 0.0	1.1	8.1	N.D	91.9
	**180**	90.6	< 0.0	1.6	7.7	N.D	92.3
	**240**	93.9	< 0.0	1.9	4.3	N.D	95.7
	**300**	95.7	< 0.0	2.6	1.7	N.D	98.3
	**360**	91.9	< 0.0	2.3	5.7	N.D	94.3
**8** 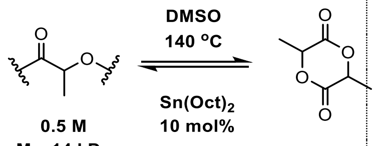 0.5 M M_n=14 kDa Đ=1.13	**10**	16.4	< 0.0	0.1	83.5	< 0.0	16.5
	**30**	46.1	< 0.0	0.8	53.0	< 0.0	47.0
	**60**	73.2	< 0.0	3.0	23.7	0.1	76.3
	**120**	85.7	0.1	5.7	8.3	0.1	91.7
	**180**	88.4	0.2	7.9	3.2	0.3	96.8
	**240**	86.0	0.3	9.2	4.2	0.3	95.8
	**300**	83.4	0.5	13.2	2.5	0.3	97.5
**9** 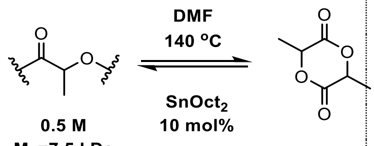 0.5 M M_n=7.5 kDa Đ=1.73	**10**	23.3	< 0.0	0.3	76.4	N.D	23.6
	**30**	55.3	< 0.0	0.2	44.5	N.D	55.5
	**60**	76.5	< 0.0	0.6	22.9	N.D	77.1
	**120**	89.6	< 0.0	1.1	9.3	N.D	90.7
	**180**	91.1	< 0.0	1.3	7.5	N.D	92.5
	**240**	91.0	< 0.0	2.0	7.0	N.D	93.0
**10** 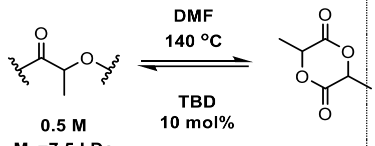 0.5 M M_n=7.5 kDa Đ=1.73	**1**	61.6	0.9	15.0	22.4	N.D	77.6
	**4**	62.3	1.3	17.7	18.7	N.D	81.3
	**6**	63.5	1.1	16.7	18.7	N.D	81.3
	**8**	63.4	1.1	16.7	18.8	N.D	81.2
	**10**	62.6	1.1	16.7	19.6	N.D	80.4
	**15**	62.2	1.1	16.2	20.6	N.D	79.4
**11** 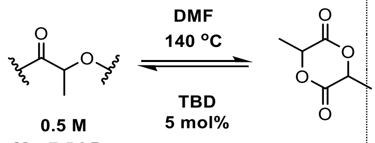 0.5 M M_n=7.5 kDa Đ=1.73	**1**	52.0	1.0	14.4	32.6	N.D	67.4
	**4**	54.2	1.0	14.6	302	N.D	69.8
	**6**	53.9	1.0	14.9	30.2	N.D	69.8
	**8**	55.6	1.2	16.3	26.8	N.D	73.2
	**10**	54.8	1.2	16.0	28.0	N.D	72.0
	**15**	55.1	1.0	15.0	28.8	N.D	71.2

Reaction	Time (min)	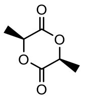 LLA (%)	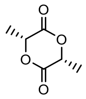 DLA (%)	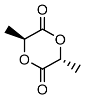 meso-LA (%)	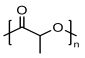 PLA (%)	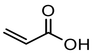 Acrylic acid (%)	Total conv. (%)
**12** 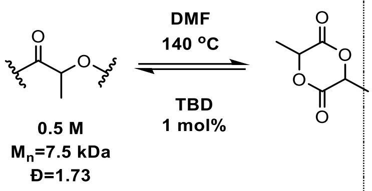	**1**	2.7	< 0.0	0.2	97.1	N.D	2.9
	**2**	2.8	< 0.0	0.3	69.9	N.D	3.1
	**4**	2.8	< 0.0	0.4	96.8	N.D	3.2
	**6**	2.9	< 0.0	0.2	96.9	N.D	3.1
	**8**	3.2	< 0.0	0.3	96.4	N.D	3.6
	**10**	3.4	< 0.0	0.4	96.2	N.D	3.8
	**15**	3.8	< 0.0	0.5	95.6	N.D	4.4
	**120**	9.1	0.1	1.8	89.0	N.D	11.0
	**180**	11.7	0.1	2.5	85.7	N.D	14.3
**13** 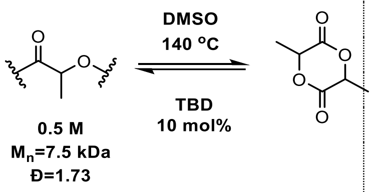	**10**	51.2	0.9	13.5	34.4	N.D	65.6
	**30**	54.7	1.0	14.8	29.6	N.D	70.4
	**60**	55.0	1.0	14.5	29.5	N.D	70.5
	**120**	49.3	0.9	13.5	36.3	N.D	63.7
**14** 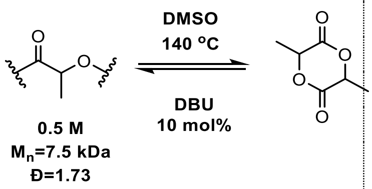	**10**	37.8	2.1	18.0	42.1	N.D	57.9
	**30**	44.6	1.6	16.9	36.9	N.D	63.1
	**60**	49.1	1.9	19.3	29.7	N.D	70.3
	**120**	43.5	0.9	12.4	43.2	N.D	56.8
**15** 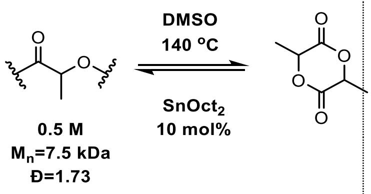	**10**	10.2	< 0.0	0.2	89.5	< 0.0	10.5
	**30**	30.8	< 0.0	0.4	68.8	< 0.0	31.2
	**60**	52.9	< 0.0	1.0	46.1	< 0.0	53.9
	**120**	76.3	< 0.0	3.3	20.2	0.1	79.8
	**180**	80.9	0.1	5.4	13.4	0.2	86.6
**16** 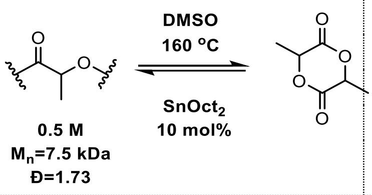	**10**	15.1	< 0.0	0.1	84.8	< 0.0	15.2
	**30**	58.7	< 0.0	1.8	39.4	0.1	60.6
	**60**	79.7	0.1	5.2	14.6	0.4	85.4
	**120**	77.2	0.2	7.7	13.6	1.3	86.4
**17** 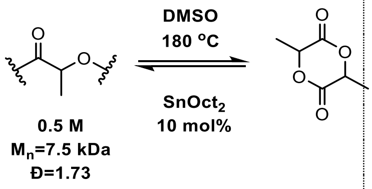	**1**	0.6	< 0.0	< 0.0	99.4	< 0.0	0.6
	**10**	25.5	< 0.0	0.3	74.2	0.3	25.8
	**30**	74.6	0.1	4.2	20.7	4.2	79.3
	**60**	81.0	0.2	7.3	10.5	7.3	89.5
	**120**	62.1	0.1	5.9	27.4	5.9	72.6
	**240**	76.1	0.2	7.4	15.1	7.4	84.9

Reaction	Time (min)	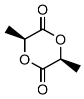 LLA (%)	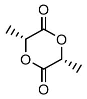 DLA (%)	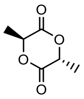 meso-LA (%)	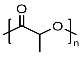 PLA (%)	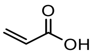 Acrylic acid (%)	Total conv. (%)
**18** 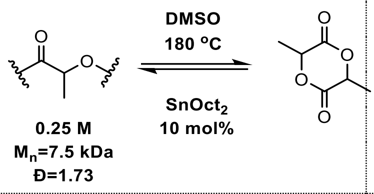	**10**	30.1	< 0.0	0.5	69.4	< 0.0	30.6
	**30**	74.9	< 0.0	3.5	20.8	0.8	79.2
	**60**	69.9	0.1	5.2	22.5	2.3	77.5
	**120**	44.3	1.5	1.5	42.2	11.9	57.8
**19** 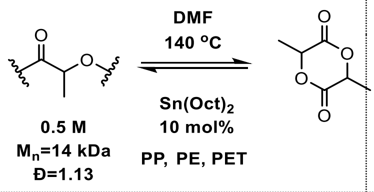	**60**	89.1	< 0.0	0.9	10.0	N.D.	90.0
	**90**	92.0	< 0.0	1.2	6.7	N.D.	93.3

*Reaction **6**: represents the total amount of LLA+DLA. The DLA content could not be calculated since the polymer contains 3-5 % DLA from the beginning, and equation **(SE3)** is only valid for depolymerization of pure PLLA.[14]

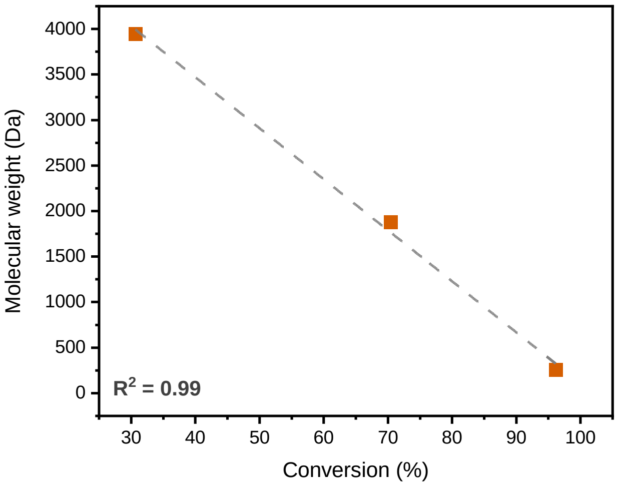

Supporting Fig. 5. Decrease in molecular weight in relation to polymer conversion. *Data from PLLA (M_n = 14,000 g mol^{-1}, Đ = 1.13) depolymerization in DMF (0.5 M) at 140 °C with 10 mol% Sn(Oct)$_2$. The molecular weight was determined by end-group analysis of ^{1}H NMR spectra.*

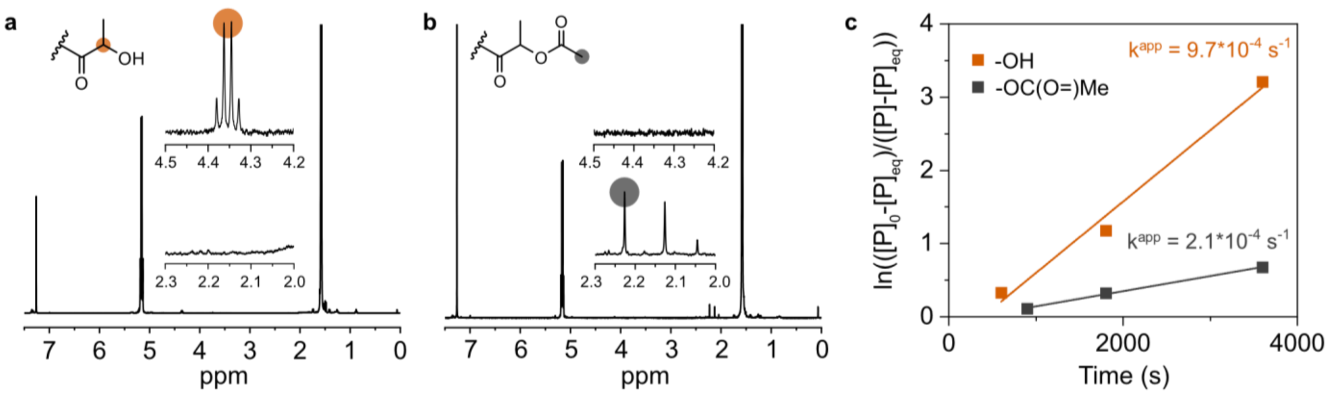

Supporting Fig. 6. End capping and subsequent depolymerization. *a) ^{1}H NMR before end capping. PLLA (M_n = 14,000 g mol^{-1}; Đ = 1.13) with -OH end group functionality (PLLA-OH). b) ^{1}H NMR after end capping. PLLA with -OC(=O)Me end group functionality (PLLA-OC(=O)Me). c) Depolymerization rate in relation to end group functionality. Data from reaction performed in DMF (0.5 M calculated on LLA repeating unit) at 140 °C with 10 mol% Sn(Oct)$_2$. The lower depolymerization rate of PLLA-OC(=O)Me supports that the depolymerization takes place through an "unzipping" mechanism from the OH functional chain end.*

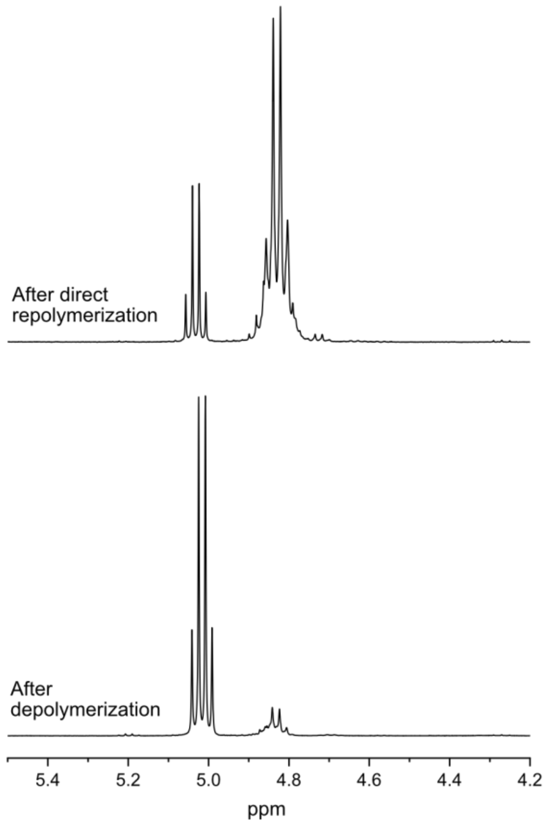

Supporting Fig. 7. Direct repolymerization. *Depolymerization of PLLA (14,000 g mol^{-1}) in DMF (0.5 M calculated on the PLLA repeating unit) at 140 °C, followed by direct repolymerization of crude LLA in DMF by addition of 10 mol% TBD. The polymerization was performed at RT for 24 h. The equilibrium conversion to polymer was 83%, which can be compared to the theoretical equilibrium conversion to polymer at 20 °C (82% at 0.5 M concentration in DMF; calculated from thermodynamic data in Supporting Tab. 3). Hence, the formed LLA is active for ROP and exhibits the same equilibrium behavior as is expected for neat LLA. However, ^{1}H NMR end group analysis suggests a new molecular weight of around 3,000 g mol^{-1}.*

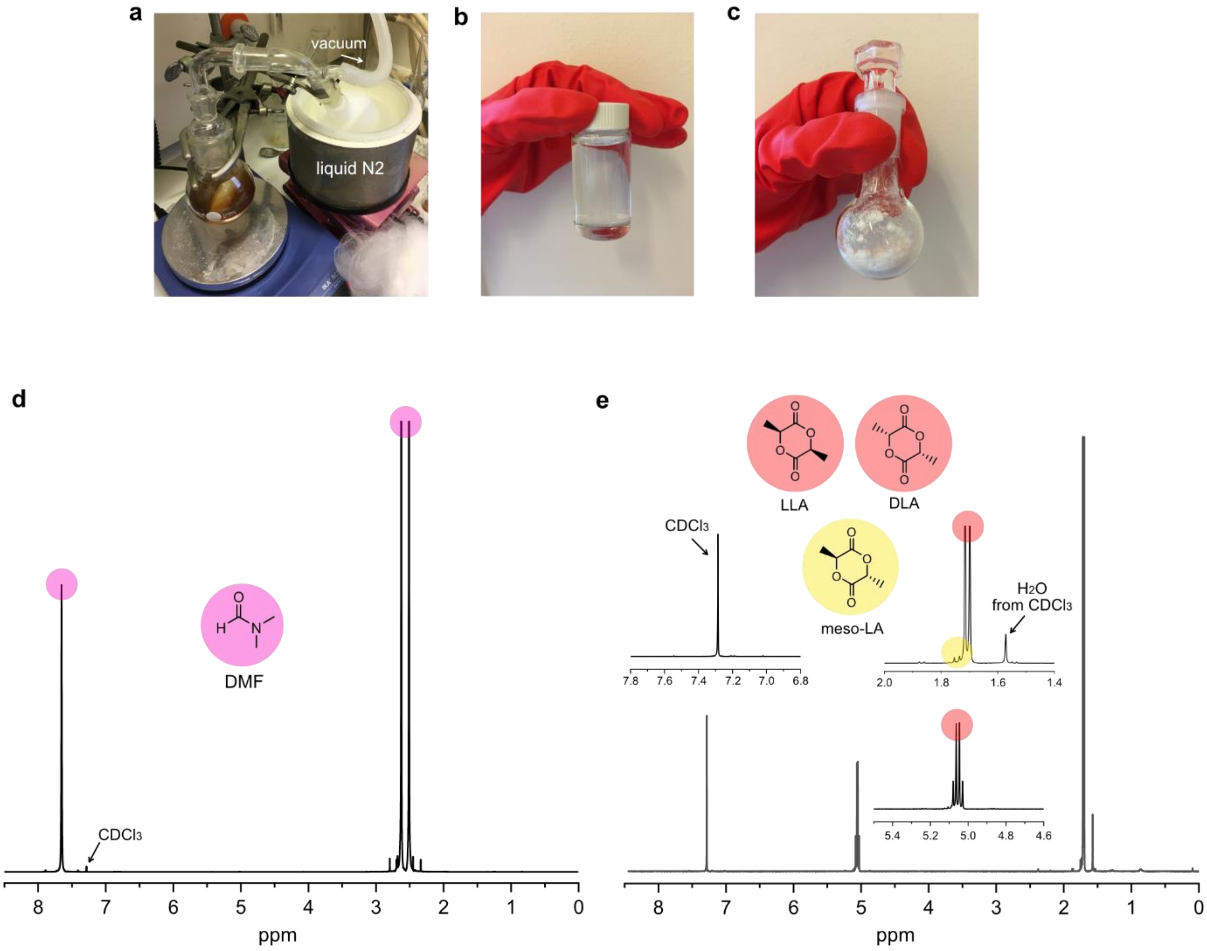

Supporting Fig. 8. Isolation of recycled LA. *a) Setup for distillation of DMF. b) DMF recovered after distillation (84% yield). c) Recycled and isolated LA (38% yield). d) ^{1}H NMR of recovered DMF (99.9% purity). e) ^{1}H NMR of recycled and isolated LA (meso-LA content: 3%).*

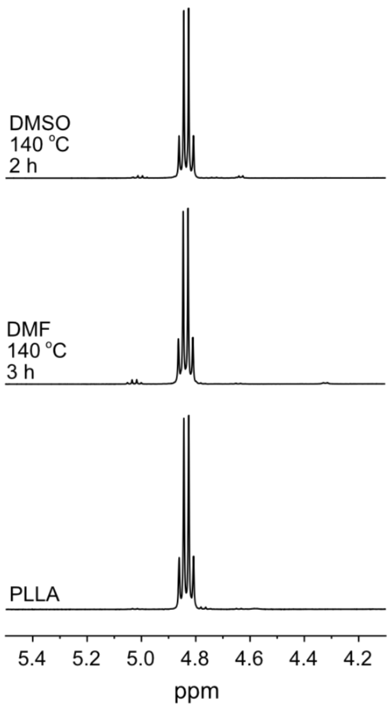

Supporting Fig. 9. Catalytic activity of solvents. *To investigate the catalytic activity of the solvents, PLLA (M_n = 14,000 g mol^{-1}; Đ = 1.13) dissolved in DMF and DMSO (0.5 M) were heated at 140 °C for at least 2 h. ^{1}H NMR revealed no ring-closing depolymerization during this period of time, indicating that the solvents have no catalytic activity toward ring-closing depolymerization at this temperature.*

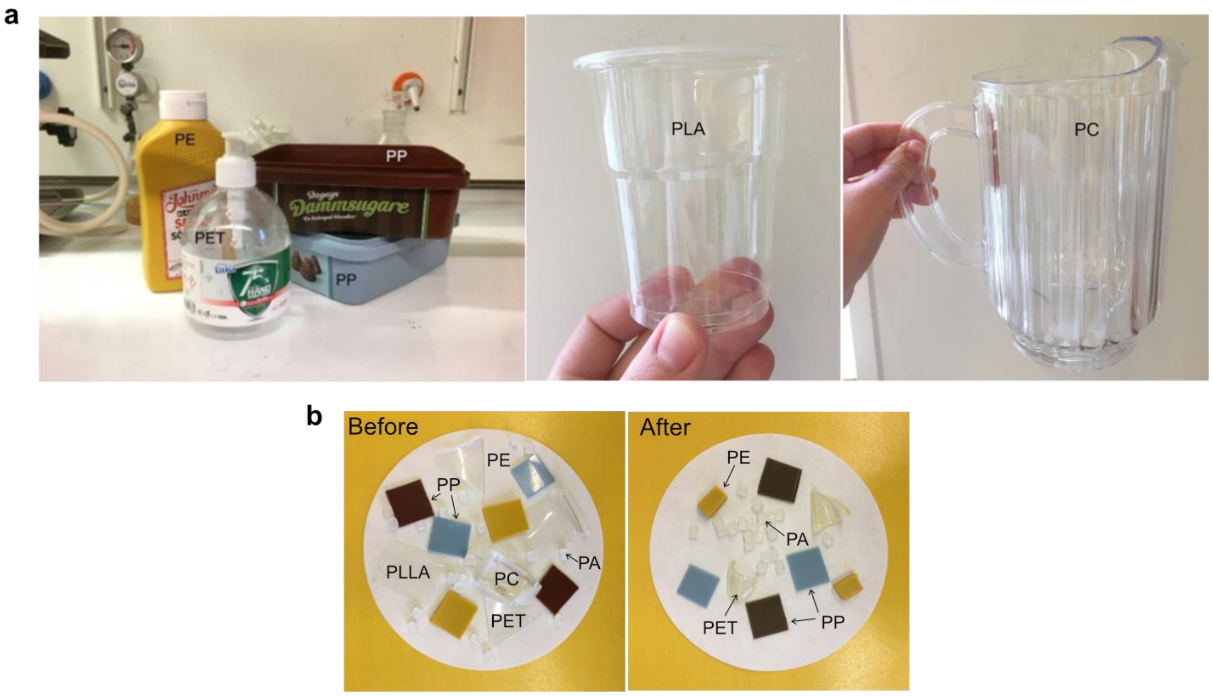

Supporting Fig. 10. PLA recycling in the presence of mixed plastic waste. *a) Images of the six post consumer plastics used in the experiment. The PE flask used to contain mustard, the PP boxes cookies and PET bottle hand sanitizer. b) Images of plastic pieces before and after depolymerization.*

Polymerization thermodynamics

Supporting Tab. 3. Data for determination of polymerization thermodynamic parameters. *All polymerizations were performed at $[M]_0$ = 0.5 M, with 10 mol% DBU as catalyst and monomer:initiator feed ratio [M]:[I] = 100:1.*

Solvent	Temperature (°C)	Equilibrium converison (%)
DMF	65	56.4
DMF	75	46.9
DMF	85	37.3
DMF	95	29.3
GVL	55	74.4
GVL	65	69.7
GVL	75	62.8
GVL	95	47.9
DMSO	65	39.0
DMSO	75	33.1
DMSO	85	26.8
DMSO	95	21.0
DX	55	87.2
DX	65	85.3
DX	75	82.9
DX	85	79.6
PhCl	35	98.3
PhCl	65	96.7
PhCl	95	93.3
PhCl	125	89.3

Supporting Note 2. Calculation of polymerization thermodynamic parameters. *The Dainton-Ivin[15] equation defines the relationship between polymerization temperature T, ΔH_p, ΔS_p and the monomer equilibrium concentration $[M]_{eq}$, were R is the gas constant:*

$$T = \frac{\Delta H_p}{\Delta S_p + R\ln[M]_{eq}}$$

(SE9)

Under the assumption that the volume does not change upon polymerization, the relationship can be expressed in terms equilibrium molar fraction $[M]_{eq}/[M]_0$, rather than $[M]_{eq}$:

$$T = \frac{\Delta H_p}{\Delta S_p + R\ln([M]_{eq}/[M]_0)} \qquad \textbf{(SE10)}$$

The temperature at which $[M]_{eq} = [M]_0$ is defined as the ceiling temperature T_c:

$$T_c = \frac{\Delta H_p}{\Delta S_p}$$

(SE11)

*Further, equation **SE10** can be rearranged to:*

$$R\ln([M]_{eq}/[M]_0) = \frac{1}{T} * \Delta H_p - \Delta S_p \qquad \textbf{(SE12)}$$

In this from, the Dainton-Ivin equation can be used to calculate ΔH_p and ΔS_p via linear regression of $R\ln([M]_{eq}/[M]_0)$ as a function of 1/T. The equation can also be rearranged to express the equilibrium molar fraction as a function of T when ΔH_p and ΔS_p are known:

$$[M]_{eq}/[M]_0 = e^{\frac{\Delta H_p}{RT}-\frac{\Delta S_p}{R}} \qquad \textbf{(SE13)}$$

Supporting Note 3. Polymerization thermodynamics for short linear chains. *Equation **SE9** is based on Flory's assumption that the reactivity of the propagating end becomes independent of molecular weight after a certain macromolecular chain length. Disregarding this assumption, the degree of polymerization DP_n should be considered. Thus, **SE9** can be rewritten accordingly:*[16]

$$T = \frac{\Delta H_p}{\Delta S_p + R\ln\left(\frac{DP_n}{DP_n - 1}[M]_{eq}\right)} \qquad \textbf{(SE14)}$$

Further, the equation can be rearranged to:

$$[M]_{eq} = \frac{DP_n - 1}{DP_n} e^{\frac{\Delta H_p}{RT}-\frac{\Delta S_p}{R}} \qquad \textbf{(SE15)}$$

*For long linear chains, the term $(DP_n - 1)/DP_n$ will be approaching 1 and can, therefore, be neglected (Flory's assumption). However, for shorter oligomeric chains (approx. $DP_n \leq 20$) the effect of DP_n on the thermodynamic equilibrium between polymer and monomer is noteworthy, and the $[M]_{eq}$ will decrease with decreasing DP_n. This explains the observed decline in conversion of polymer to monomer with decreased feed molecular weight (**Supporting Tab. 1.1-1.3**).*

Supporting Tab. 4. Relationship between depolymerization behaviour and polymerization thermodynamics. *All depolymerizations were performed at 65 °C with $[M]_0$ = 0.5 M and 10 mol% DBU as catalyst. The predicted values were calculated from ΔH_p and ΔS_p in each solvent according to equation **SE13**.The experimental values were determined from the ^{1}H NMR C**H** peaks for LLA/DLA and meso-LA/PLA:*

$$[M]_{eq}/[M]_0 = \frac{\frac{\int CH(LLA/DLA)}{L+D}}{\int CH(LLA/DLA) + \int CH(meso-LA/PLA)} \qquad \textbf{(SE16)}.$$

**Solvent**	**Experimental $[M]_{eq}/[M]_0$ (%)**	**Predicted $[M]_{eq}/[M]_0$ (%)**
DMF	43.5	44.0
GVL	27.0	30.2
DMSO	54.1	60.9
DX	17.3	15.0
PhCl	3.30	3.50

Relationship between solubility parameters and T_c

Supporting Note 4. Variation in T_c with different solvents. *The ceiling temperature T_c is determined according to equation **SE11**. We are interested in how T_c varies in solvent A compared to solvent B:*

$$\Delta T_c = T_c^A - T_c^B = \frac{\Delta H_p^A}{\Delta S_p^A} - \frac{\Delta H_p^B}{\Delta S_p^B} \tag{SE17}$$

We can write the free energy of polymerization ΔG_p, hence also ΔH_p and ΔS_p, as a sum of several contributions – the actual chemical reaction, an ideal part (changes in entropy of mixing), and the direct effects from the solvent (interactions and entropic effects due to restructuring around the monomer/polymer):

$$\Delta G_p = \Delta G_{react} + \Delta G_{ideal} + \Delta G_{solv} \tag{SE18}$$

$$\Delta T_c = \frac{\Delta H_{react}^B + \Delta H_{ideal}^B + \Delta H_{solv}^B}{\Delta S_{react}^B + \Delta S_{ideal}^B + \Delta S_{solv}^B} - \frac{\Delta H_{react}^A + \Delta H_{ideal}^A + \Delta H_{solv}^A}{\Delta S_{react}^A + \Delta S_{ideal}^A + \Delta S_{solv}^A} \tag{SE19}$$

All contribution from the chemical reaction and the ideal contribution are solvent independent. Then how about the solvent contribution? The change in free energy of solvation upon polymerization, ΔG_{solv}, can be written as the difference between the solvation energy of the polymer and the solvation energy of the monomer:

$$\Delta G_{solv} = \Delta G_{solv}^p - \Delta G_{solv}^m \tag{SE20}$$

*The solvation energies of LLA and PLLA were calculated from molecular dynamics (MD) simulations as a function of temperature. Since, ΔS_{solv} of both LLA and PLLA are solvent independent (Fig. 7a-b. in article), equation **SE19** can be rewritten accordingly, which leads to T_c being proportional to ΔG_{solv}:*

$$\Delta T_c = \frac{\Delta H_{solv}^B - \Delta H_{solv}^A}{\Delta S_p} = \frac{\Delta G_{solv}^B - \Delta G_{solv}^A}{\Delta S_p} \rightarrow T_c \propto \Delta G_{solv} \tag{SE21}$$

This is supported by the experimentally determined T_c that has a linear relationship ΔG_{solv} (Fig. 5c. in main article). Thus, MD simulations can be used to predict differences in T_c between different solvents. However, a tool that is simpler than MD simulations would be useful for screening and to predict which solvents might be suitable to promote ring-closing depolymerization.

According to the Ivin-Léonards theory,[17] ΔG_p can be written as a function of the Flory-Huggins interaction parameter of monomer-solvent, solvent-polymer and monomer-polymer (χ_{ms}, χ_{sp} and χ_{mp}; φ_m, φ_p and φ_s denotes volume fractions of monomer, polymer and solvent; V_m and V_s denotes molar volumes of monomer and solvent):

$$\Delta G_p = RT\left(ln\,\phi_m + 1 + \left(\chi_{ms} - \chi_{sp}(V_m/V_s)\right)\phi_s + \chi_{mp}(\phi_p - \phi_m)\right) \tag{SE22}$$

Since χ_{mp} is solvent independent, the solvent effect on the monomer-polymer equilibrium can be summarized in the term:

$$\beta = \chi_{ms} - \chi_{sp}(V_m/V_s) \tag{SE23}$$

The difference in ΔG_p between solvent A and B, at a constant φ_s, can be written accordingly:

$$\Delta G_p^B - \Delta G_p^A = RT(\beta^B - \beta^A)\phi_s \tag{SE24}$$

*Combining equation **SE24** with **SE21**, it becomes clear that T_c is proportional to β:*

$$\Delta T_c = \frac{(\beta^B - \beta^A)\phi_s}{\Delta S_p} \rightarrow T_c \propto \beta \qquad \textbf{(SE25)}$$

While χ_{ms} and χ_{sp} have to be determined for each solvent experimentally, the Hildebrand solubility parameters δ_m, δ_p and δ_s can easily be obtained from tables or physical data found in literature. In contrast to the Flory-Huggins interaction parameter, which contains both an enthalpic and an entropic contribution, the Hildebrand solubility parameter only counts for the enthalpic contribution. However, MD simulations showed that the differences in solvation entropy between different solvents were neglectable (Fig. 5 a-b. in main article), why the difference in T_c can be assumed to be an entopic effect alone. Hence, it is likely that the Hildebrand solubility parameter can be used for the purpose of screening and to predict which solvents should be the suitable to promote ring-closing depolymerization:

$$\chi_{ms} \propto (\delta_m - \delta_s)^2 \; and \; \chi_{sp} \propto \left(\delta_s - \delta_p\right)^2 \rightarrow T_c \propto (\delta_m - \delta_s)^2 \; and \; \left(\delta_s - \delta_p\right)^2 \qquad \textbf{(SE26)}$$

Supporting Tab. 5. Hansen's solubility parameters. *Physical data and Hansen's solubility parameters δ calculated according to equation **SE27**. The temperature T = 298 K was used for all calculations, and R denotes the gas constant:*

$$\delta = \sqrt{\frac{H_{vap} - RT}{M/\rho}} \qquad \textbf{(SE27)}$$

**Substance**	**ΔH_{vap} ($kJ*mol^{-1}$)**	**ρ ($g*mL^{-1}$)**	**M ($g*mol^{-1}$)**	**δ ($MPa^{1/2}$)**
DMF	46.9[18]	0.94[19]	73.1	24.0
GLV	54.8[18]	1.05[20]	100.1	23.4
DMSO	52.9[18]	1.10[19]	78.1	26.6
DX	38.6[18]	1.03[19]	88.1	20.5
PhCl	41.2[18]	1.11[21]	112.6	19.5
LLA	69.3[22]	1.2	144.1	23.6
PLA	-	-	-	20.2[23]

Supporting Tab. 6. Relationship between solubility parameters and T_c. *Multiple linear regression input and summary of results. The ceiling temperatures T_c were calculated according to equation* ***SE11****, based on values of ΔH_p and ΔS_p determined via equation* ***SE12****. $(\delta_m-\delta_s)^2$ and $(\delta_s-\delta_p)^2$ were calculated from values in* ***Supporting Tab. 5,*** *where the subscripts denote monomer (m), polymer (p) and solvent (s). The multiple linear regression was performed using the data analysis regression function in Microsoft Excel, at a confidence level of 95 %.*

INPUT

Solvent	T_c (K)<br>y	$(\delta_m-\delta_s)^2$<br>x_1	$(\delta_s-\delta_p)^2$<br>x_2
DMF	390.8	0.138	14.1
GVL	410.3	0.0263	10.4
DMSO	398.9	9.03	40.9
DX	550.2	9.31	0.113
PhCl	615.3	16.4	0.434

RESULTS

R^2 = 0.998

	Coefficient	P-value
**Intercept**	444	0.00015
**x_1**	10.8	0.00196
**x_2**	-3.53	0.00318

Molecular modelling

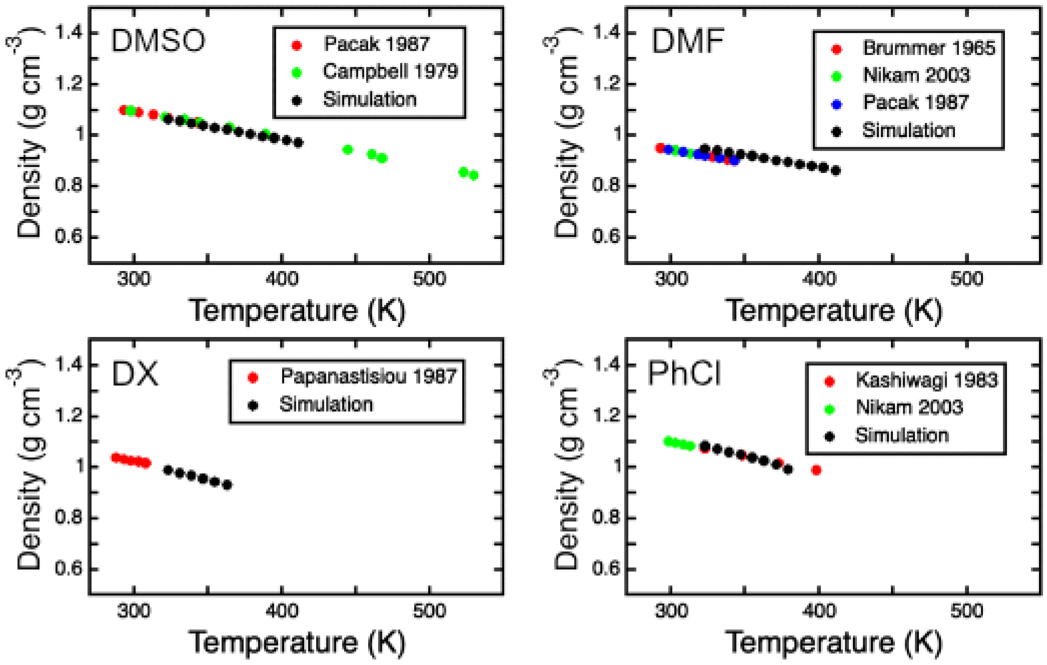

Supporting Fig. 11. Liquid densities as a function of temperature. *Experimental data from Pacak*[24] *(DMSO and DMF); Campbell*[25] *(DMSO); Brummer*[26] *(DMF); Nikam and Kharat*[27] *(DMF and Chlorobenzene); Papanastasiou et al.*[28] *(DX)*

Supporting references

[1] M. J. Abraham, T. Murtola, R. Schulz, S. Páll, J. C. Smith, B. Hess, E. Lindahl, *SoftwareX* **2015**, *1*, 19–25.
[2] G. Bussi, D. Donadio, M. Parrinello, *J. Chem. Phys.* **2007**, *126*, 14101.
[3] T. Darden, D. York, L. Pedersen, *J. Chem. Phys.* **1993**, *98*, 10089–10092.
[4] U. Essmann, L. Perera, M. L. Berkowitz, T. Darden, H. Lee, L. G. Pedersen, *J. Chem. Phys.* **1995**, *103*, 8577–8593.
[5] M. Parrinello, A. Rahman, *J. Appl. Phys.* **1981**, *52*, 7182–7190.
[6] B. Hess, *J. Chem. Theory Comput.* **2008**, *4*, 116–122.
[7] Y. Sugita, Y. Okamoto, *Chem. Phys. Lett.* **1999**, *314*, 141–151.
[8] K. Vanommeslaeghe, E. Hatcher, C. Acharya, S. Kundu, S. Zhong, J. Shim, E. Darian, O. Guvench, P. Lopes, I. Vorobyov, *J. Comput. Chem.* **2010**, *31*, 671–690.
[9] K. Vanommeslaeghe, A. D. MacKerell Jr, *J. Chem. Inf. Model.* **2012**, *52*, 3144–3154.
[10] K. Vanommeslaeghe, E. P. Raman, A. D. MacKerell Jr, *J. Chem. Inf. Model.* **2012**, *52*, 3155–3168.
[11] T. P. Straatsma, J. A. McCammon, *Annu. Rev. Phys. Chem.* **1992**, *43*, 407–435.
[12] C. H. Bennett, *J. Comput. Phys.* **1976**, *22*, 245–268.
[13] Q. Yang, M. Sheng, Y. Huang, *Org. Process Res. Dev.* **2020**, *24*, 1586-1601.
[14] L. Feng, X. Chen, X. Bian, S. Xiang, B. Sun, Z. Chen, *Chemom. Intell. Lab. Syst.* **2012**, *110*, 32-37.
[15] F. S. Dainton, K. J. Ivin, *Nature* **1948**, *162*, 705-707.
[16] J. Libiszowski, A. Kowalski, R. Szymanski, A. Duda, J.-M. Raquez, P. Degée, P. Dubois, *Macromolecules* **2004**, *37*, 52-59.
[17] K. J. Ivin, J. Léonard, *Eur. Polym. J.* **1970**, *6*, 331-341.
[18] W. Acree Jr, J. S. Chickos, *J. Phys. Chem. Ref. Data* **2016**, *45*, 33101.
[19] T.M. Aminabhavi, V. B. Patil, *J. Chem. Eng. Data* **1998**, *43*, 497-503.
[20] Z. S. Baird, P. Uusi-Kyyny, J.-P. Pokki, E. Pedegert, V. Alopaeus, *Int. J. Thermophys.* **2019**, *40*, 1-36.
[21] H. Kashiwagi, T. Fukunaga, Y. Tanaka, H. Kubota, T. Makita, *J. Chem. Thermodyn.* **1983**, *15*, 567-580.
[22] V. N. Emel'Yanenko, S. P. Verevkin, A. A. Pimerzin, *Russ. J. Phys. Chem. A* **2009**, *83*, 2013-2021.
[23] D. Karst, Y. Yang, *J. Appl. Polym. Sci.* **2005**, *96*, 416-422.
[24] P. Pacak, *J. Solution Chem.* **1987**, *16*, 71-77.
[25] A. N. Campbell, *Can. J. Chem.* **1979**, *57*, 705-707.
[26] S. B. Brummer, *J. Chem. Phys.* **1965**, *42*, 1636-1646.
[27] P. S. Nikam, S. J. Kharat, *J. Chem. Eng. Data* **2003**, *48*, 1202-1207.
[28] G. E. Papanastasiou, A. D. Papoutsis, G. I. Kokkinidis, *J. Chem. Eng. Data* **1987**, *32*, 377-381.
